# Nitrogen Fertilization Effects on Soil Bacterial Communities, Nitrogen-Cycling Genes, and Wheat Yield Across Different Soil Types in the North China Plain

**DOI:** 10.3390/microorganisms13102382

**Published:** 2025-10-15

**Authors:** Geng Ma, Xiaoyan Zhang, Xiaojie Han, Juan Kang, Haiyan Zhang, Yanfei Zhang, Hongfang Lu, Yingxin Xie, Dongyun Ma, Chenyang Wang

**Affiliations:** 1 College of Agronomy, Henan Agricultural University, Zhengzhou 450002, China; nymg5135@163.com (G.M.);; 2 College of Resource and Environmental Sciences, Henan Agricultural University, Zhengzhou 450002, China; 3 National Engineering Research Centre for Wheat, Henan Agricultural University, Zhengzhou 450002, China

**Keywords:** nitrogen fertilization, soil types, bacterial community, wheat yield, nitrogen functional genes

## Abstract

Nitrogen (N) fertilization is known to influence soil microbial communities and crop yield, but how N affects the bacterial community and the link to crop yield across different soil types remains poorly understood. Here, we conducted three 5-year stationary field experiments to explore the effect of N fertilization (0, 180, 240, and 300 kg ha^−1^; termed N0, N1, N2, and N3, respectively) with different soil types (fluvo-aquic soil, FS; sandy soil, SS; lime concretion black soil, BS) on bacterial communities and the relationships among soil, microbes, and N-cycling functional genes to further investigate the effects on wheat yield. The results showed that the N2 treatment (240 kg ha^−1^) achieved the highest wheat yield, with significantly lower yields in SS than those in FS and BS. N fertilization significantly altered soil physicochemical properties, with a notable decrease in pH, particularly in SS, and an increase in NO_3_^−^-N content. Bacterial α-diversity significantly decreased with N application in SS but not in FS and BS, and NO_3_^−^-N played a primary role in shaping beta diversity in FS and BS. There were 43, 62, and 11 bacterial species that changed significantly from phylum to genus in the FS, SS, and BS, respectively. The abundance of nitrification genes increased with N fertilization in FS and SS, and N-cycling genes were significantly associated with soil properties. Partial Least Squares Path Modeling (PLS-PM) revealed that N fertilizer affected soil properties, which in turn regulated bacterial communities, and ultimately influenced wheat yield, explaining 67.4% of the yield variation. This study highlights the soil-specific responses to N application, providing a basis for optimizing N management and enhancing agricultural sustainability.

## 1. Introduction

Wheat, the world’s third most cultivated cereal, faces growing demand as the Food and Agriculture Organization (FAO) predicts a global population of 9.6 billion by 2050, requiring a 60% increase in food production (FAO, 2018). Nitrogen (N) fertilizers, especially urea, have played a pivotal role in enhancing crop yields and preserving soil fertility over recent decades [1]. Ensuring food security for a growing population has led to consistent annual increases in agricultural N fertilizer application across China [2,3]. According to the National Bureau of Statistics (http://data.stats.gov.cn/ accessed on 16 December 2024), the average annual consumption of chemical N fertilizer in agriculture in China from 2015 to 2023 reached more than 19 million tons because of the notable effect of N on achieving high yield. However, the large input of N fertilizer in agricultural systems greatly exceeds the demand of the crops, which not only reduces the efficiency of N utilization but also contributes to environmental degradation, including greenhouse gas emissions, eutrophication, and soil acidification [4,5,6].

It is widely recognized that soil microbiota impact plant development and resource utilization efficiency, and play important roles in the biogeochemical cycle and the sustainability of agricultural systems [7]. Understanding how nitrogen fertilizer influences the composition, function, and dynamics of soil microbial communities is crucial for developing effective microbiome management strategies to enhance the sustainability of crop production [8]. However, the effects of N fertilizers on microbial communities and function remain inconclusive due to variations in unique initial soil characteristics, soil aeration conditions, and soil physicochemical properties across different soil types [9,10,11]. For instance, the abundance of Gammaproteobacteria in clay soil was markedly higher than in loam and loamy-sand soils [12]. Alluvial soils could suffer marked increases in ammonia content caused by unique microorganisms in response to N fertilization, and clay soils could protect the microbial biomass [13]. Moreover, N fertilizers reduce bacterial richness and alter bacterial community composition, structure, and predicted functions in tomato bulk soil [14] and in the rhizosphere soil of winter wheat [15]. In maize plants, bacterial diversity in bulk and rhizosphere soils remained unchanged after N application [16]. Yao et al. indicated that N fertilizer enhances bacterial community richness and diversity in coastal salt-affected agro-ecosystems [17]. In terms of functionality, N fertilization typically enhances denitrification potential by increasing NO_3_^−^-N, but the magnitude depends on soil aeration. In clay soils, N input significantly increased the abundance of denitrification genes, but showed no significant increase in sandy soils [18]. N fertilizer application greatly reduces the contribution of biological nitrogen fixation to cereal N uptake [19]. However, the specific impact of N fertilization on the taxonomic groups linked with diversity changes and their potential role in soil nutrient cycling remains unclear, and such inconsistencies might be caused by differences in fertilizer application rate, management regimes, and soil conditions [20]. Further studies are needed to explore how soil bacterial communities and function respond to different nitrogen fertilizer rates under various soil conditions.

Soil N availability depends on different N conversion reactions, which are performed by a complex network of microorganisms with a variety of metabolic functions [21]. The N cycle is an important part of the biogeochemical cycle, and it mainly involves nitrate reduction, denitrification, N fixation, and nitrification driven by various bacteria, archaea, and fungi [22,23]. N application has been shown to enhance the abundance of microorganisms involved in the N cycle, including ammonia-oxidizing bacteria and denitrifiers [24,25,26]. A meta-analysis of 33 field investigations demonstrated that the abundance of ammonia-oxidizing archaea (AOA) and ammonia-oxidizing bacteria (AOB) showed a positive response to N addition [27]. The *nirS* or *nirK* gene is a rate-limiting step in denitrification, encoding nitrite reductase responsible for reducing NO_2_^−^ to NO [28]. Shang et al. found that long-term (NH_4_)_2_SO_4_ addition to agricultural soils reduced pH and decreased the abundance of denitrification genes (*nirS*, *nirK*, *nosZ*) [26]. However, a meta-analysis revealed that the abundances of *nirS* and *nirK* significantly increased under N addition treatments [29]. The application of N fertilization can influence the occurrence and regulation of the dissimilatory nitrate reduction to ammonium (DNRA) process, a process increasingly recognized for its role in improving N use efficiency [30]. Li et al. revealed that high N fertilization significantly increased the relative abundance of *nifH* genes involved in N fixation and decreased that of amoA-archaea involved in ammonia oxidation, *nirS* genes involved in nitrite reduction, and *nosZ* genes involved in nitrous oxide reduction in paddy reclaimed [31]. These indicate that the responses of bacterial functions to N application vary in diverse soils. Therefore, clarifying response patterns of N-cycle genes to the long-term N application across different soil types is critical for disentangling the potential links between bacterial functions, soil properties, and crop yield under N applications.

Although the influence of N fertilizer on the assemblage and function of soil microbial communities has been widely investigated separately, the impacts of N application under varying soil conditions on soil microbial communities, functions, and underlying mechanisms that drive crop productivity have been less well studied. These findings will enable the development of effective management strategies and the engineering of synthetic microbiomes, advancing sustainable agriculture. In this study, we investigated the role of microbial community structure and function in influencing wheat productivity at five-year experimental stations that used the same treatments across three soil types. Therefore, our aims were to clarify (1) how nitrogen fertilization affects wheat yield and soil characteristics, (2) the responses of bacterial communities and N-cycling genes to N fertilization, and (3) the relationships of bacterial communities and functions to soil properties, and their potential effects on wheat yield across varying soil types.

## 2. Materials and Methods

### 2.1. Sites Description and Experimental Design

From 2013 to 2018, three experiments were conducted concurrently at Xuchang (33°58′ N, 113°55′ E), Kaifeng (34°38′ N, 114°55′ E), and Shangshui (33°33′ N, 114°37′ E) in Henan Province, China. Experimental plots were established at all three sites in 2013, and annual mean temperatures were 14.5 °C, 14.0 °C, and 14.5 °C; and the annual rainfalls were 729 mm, 650 mm, and 784 mm, respectively. These three experimental sites have similar climatic conditions but different soil conditions: fluvo-aquic soil (FS), which contains 45.83% sand, 32.26% silt, and 21.91% clay, along with a relatively high content of available potassium; sandy soil (SS), which contains 57.87% sand, 27.61% silt, and 14.52% clay, as well as a relatively low content of total nitrogen; and lime-concretion black soil (BS), which contains 12.42% sand, 47.33% silt, and 40.25% clay, along with a relatively high content of available nitrogen [32]. The basic properties of the plow layer soil (0–20 cm) are shown in Table S1.

The present study used the high-yielding wheat variety ‘Yumai 49–198’, which is planted extensively in the Huang-Huai wheat production area. Four different N fertilizer rates were used in the field experiments (0, 180, 240, and 300 kg ha^−1^; termed N0, N1, N2, and N3, respectively) in the three sites. Each plot was 6.0 m × 3.0 m (18.0 m^2^) with four replicates in a randomized design. Urea (46% N), calcium superphosphate (15% P_2_O_5_), and potassium chloride (60% K_2_O) were applied at rates of 150 kg P_2_O_5_ ha^−1^ and 120 kg K_2_O ha^−1^. Half the nitrogen and all phosphorus and potassium fertilizers were broadcast before plowing at sowing. The remaining nitrogen was top-dressed at the jointing stage in selected plots. Pest, disease, and weed management followed local high-yield wheat practices.

### 2.2. Sampling Methods and Analysis

The wheat yield was recorded each year at the three sites. Soil samples from 0 to 20 cm in the plow layer were collected from the three fertilization experimental fields in June 2018 after wheat harvest, because the soil microbial communities reached a relatively stable state after five years of concurrent experiments. From each plot, five bulk soil samples (five-point sampling method) were collected using a 5 cm diameter soil sampler and thoroughly mixed to form a single homogeneous sample, with four biological replicates conducted. In this study, 48 samples (four treatments × four repetitions × three sites) were collected. Each soil sample was divided into two subsamples: one air-dried for chemical analysis and the other sieved (2 mm mesh) and stored at −80 °C for microbial analysis. The soil sample was dried to a constant weight at 105 °C in an oven to determine the water content. Soil pH was measured potentiometrically in a 1:5 soil/water suspension by a pH meter. Organic matter (OM) and total nitrogen (TN) were quantified using dichromate oxidation. Available nitrogen (AN) was determined by alkaline hydrolysis diffusion, available phosphorus (AP) by sodium bicarbonate extraction followed by molybdenum-blue spectrophotometry, and available potassium (AK) by ammonium acetate (1 M) displacement and flame photometry. A continuous flow approach (Santt System, Skalar, Holland) was used to determine the NO_3_^−^-N content of soil extracted with 2 M KCl [33].

### 2.3. DNA Extraction from Soil, PCR Amplification, and Sequencing

Microbial DNA was extracted from soil samples (0.5 g fresh weight) using the E.Z.N.A. Soil DNA Kit (Omega Bio-Tek, Norcross, GA, USA) according to the manufacturer’s instructions. The extracted DNA concentration was determined using a NanoDrop 2000 UV-vis spectrophotometer (Thermo Fisher Scientific, Waltham, MA, USA), and its quality was assessed by 1% agarose gel electrophoresis. The V3–V4 hypervariable regions of the bacterial 16S rRNA gene were then amplified by PCR using primers 338F (5′-ACTCCTACGGGAGGCAGCAG-3′) and 806R (5′-GGACTACHVGGGTWTCTAAT-3′) [34,35]. The PCR protocol consisted of: initial denaturation at 95 °C for 3 min; 27 cycles of denaturation (95 °C for 30 s), annealing (55 °C for 30 s), and elongation (72 °C for 45 s); followed by a final elongation at 72 °C for 10 min; and a hold at 10 °C. Reactions were performed in triplicate in a 20 µL volume containing 10 ng template DNA, 4 µL of 5 × FastPfu Buffer, 2 µL of 2.5 mM dNTPs, 0.8 µL of each primer (5 µM), and 0.4 µL of FastPfu Polymerase. The resulting amplicons were separated on a 2% agarose gel, excised, and purified using an AxyPrep DNA Gel Extraction Kit (Axygen Biosciences, Union City, CA, USA), and quantified using QuantiFluor-ST (Promega, Madison, WI, USA), according to the manufacturer’s protocol. The purified amplicons were pooled at equimolar concentrations, followed by sequencing using the Illumina MiSeq platform (Illumina, San Diego, CA, USA) to produce 2 × 300 paired-end reads using standard protocols. MajorBio Bio-Pharm Technology Co., Ltd. (Shanghai, China) carried out the sequencing. The raw sequencing reads were deposited in the NCBI Sequence Read Archive (SRA) database (Accession Number: SRP266837).

### 2.4. Bioinformatics Analysis

QIIME (version 1.9.1) [36] was used to process the raw reads, with post-trimming steps integrated into the workflow. The raw fastq files were processed through demultiplexing, quality filtering using Trimmomatic, and merging using FLASH, based on the following criteria: (i) reads were truncated at sites with an average quality score below 20 using a 50 bp sliding window; (ii) reads containing ambiguous bases were removed, and primers were matched with up to two mismatches; and (iii) sequences with overlapping regions longer than 10 bp were merged based on their overlap. Prior to OTU clustering, rarefaction analysis was performed using QIIME to evaluate the rationality of sequencing depth: rarefaction curves were generated by randomly subsampling sequences from each sample until the curves reached a plateau, confirming that the sequencing depth was sufficient to capture the majority of microbial diversity in the samples. UPARSE (version 7.1) was used to cluster OTUs at a 97% similarity cutoff. After clustering, OTUs with a read count of <3 across all samples were discarded to exclude potential artifacts caused by sequencing errors. Meanwhile, UCHIME was employed to identify and remove chimeric sequences, with the following specific cutoff: chimeric sequences were defined as those with a chimeric score > 0.5 and a match similarity < 97% to the Silva (SSU132) database, and all such identified chimeric sequences were completely filtered out before subsequent analysis [37].

Taxonomic classification of 16S rRNA gene sequences was performed using a two-step strategy to balance efficiency and classification accuracy. First, initial batch classification was conducted using the RDP Classifier algorithm (version 2.13) and the Silva database, with a 70% confidence threshold [38]. This step enabled rapid taxonomic assignment of all high-quality sequences to phylum, class, order, family, and genus levels, which is suitable for large-scale sequence datasets and laid the foundation for subsequent community composition analysis. Second, to improve the resolution of taxonomic identification for key operational taxonomic units (OTUs) and reduce ambiguity in genus-level or species-level classification, the most numerous sequence from each OTU was selected as a representative and subjected to secondary taxonomic classification via BLAST (2.14.1) searching against the GenBank database. Richness and diversity indices, including OTU count, Shannon index, Chao estimator, and ACE estimator, were calculated using QIIME software. To ensure comparability across samples, sequences were normalized by randomly selecting an equal number of sequences (42,769) from each treatment. In addition, PICRUSt2 was employed to predict putative N-cycling functional genes from 16S rRNA gene sequences via a 3-step workflow [39]. Aligning 16S sequences to the Greengenes reference tree, using a hidden-state model to estimate N-related KEGG Orthologs, and filtering KOs to retain only N-process genes. Using the hsp.py script based on the KEGG ortholog (KO) database, genes and copy numbers were estimated. Aligning reads to N-KO Hidden Markov Models (HMMs), filtering hits, normalizing by gene length, and calculating copy numbers via a scaling factor from total reads and soil microbial cell counts [40].

### 2.5. Statistical Analysis

The least significant difference (LSD) at the 5% level was used as a post-hoc test after analysis of variance (ANOVA) to assess differences among treatments in soil physicochemical properties. Before conducting ANOVA, we tested the normality of the data using the Shapiro–Wilk test and the homogeneity of variance (homoscedasticity) using Levene’s test. Spearman correlation analysis was performed using SPSS version 17.0 (IBM Corp., Armonk, NY, USA) to analyze the associations between bacterial community compositions and soil physicochemical properties. For beta-diversity analysis, non-metric multidimensional scaling (NMDS) plots based on Bray–Curtis distance were generated using the metaMDS function in the Vegan package (v2.4.3). To validate the statistical significance of the observed group separation in the NMDS plots, the analysis of similarity (ANOSIM) with 999 permutations was applied to test whether the bacterial community compositions differed significantly among treatments [41]. The Mantel test was applied to assess significant effects of environmental variables on bacterial composition. We adopted a combined approach of linear discriminant analysis (LDA) and effect size measurement (LEfSe) to identify statistically different biomarkers between groups [42]. Before performing LDA, we verified the normality of the data within each group using the Shapiro–Wilk test and the homoscedasticity using Levene’s test. The effects of soil and bacterial characteristics on yield under N application were analyzed across three soil types using Partial Least Squares Path Modeling (PLS-PM). PLS-PM is a soft-modeling approach that is relatively robust to violations of normality and homoscedasticity assumptions. However, we still examined the distribution of the latent variables and residuals. We used normal probability plots to check the normality of residuals and residual plots to check the homoscedasticity. The latent variables analyzed by PLS-PM included soil characteristics, bacterial diversity, structure, N-cycling functional genes, and wheat yields. Soil characteristics included pH and NO_3_^−^-N, diversity included sobs and chao, genes included *nirK*, *nrfH*, and *anfG*. We selected NMDS1 from the NMDS based on the OTU tables as a parameter for the bacterial community structure. The predictive power of different models was evaluated using the goodness-of-fit (GOF) measure. For the PLS-PM model, a GOF value above 0.6 is considered acceptable. For the PLS-PM model, GOF values above 0.6 are acceptable.

## 3. Results

### 3.1. Wheat Yield and Soil Properties

**Figure 1:**
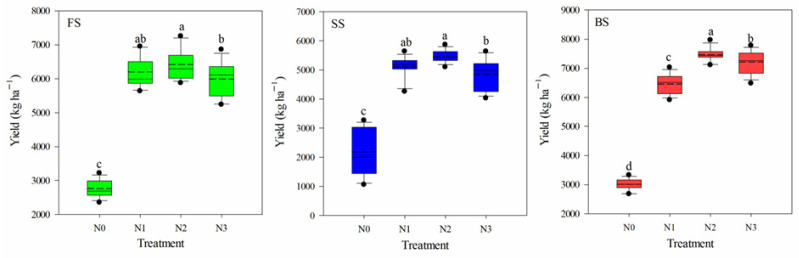
Box plots showing the wheat yields response to the application of N fertilizer at different rates in three soils. Solid and dashed lines, box boundaries, and bars and dots inside or outside of the boxes represent the median and mean values, the 25th and 75th, the 10th and 90th, and <5th and >95th percentiles of all data, respectively. Different letters above the boxes indicate significant differences among different N treatments for each soil type (*p* < 0.05).

Across the five years of the experiment (2013–2018), the average wheat yields for the SS were significantly lower than those for the BS and FS. Irrespective of soil type, wheat yield was significantly affected by the N application rate (*p* < 0.05) (Figure 1). Compared with the N0 treatment, the grain yields in the N1, N2, and N3 treatments were significantly increased by 124.5%, 132.4%, and 116.7% in the FS; by 123.9%, 139.4%, and 112.6% in the SS; and by 114.6%, 148.1%, and 139.7% in the BS, respectively. The FS and BS contained greater amounts of nutrients (TN, AN, AP, and AK) than the SS, and within an individual soil, N fertilization treatment significantly affected most of the soil properties (Table 1). Compared with the N0 treatment, N application significantly decreased the soil pH in the three soil types, particularly in the SS, where the pH ranged from 7.65 to 7.09. In addition, the NO_3_*^−^*-N of N treatments increased by 146.9%, 149.9%, and 71.0% compared with the N0 treatment in the FS, SS, and BS, respectively. N application caused an increase in soil moisture, OM, TN, and AN in the FS, but significantly decreased the moisture and OM in the SS, and the OM and TN in the BS. Under the same N application treatment, the pH of the three soils showed the order of FS > SS > BS, while the TN and AN followed the order of BS > FS > SS, and the content of AK exhibited the order of FS > BS > SS. All these differences were statistically significant (Table S2).

### 3.2. Bacterial Alpha-Diversity and Community Structures

A total of 2,469,965 high-quality 16S rRNA gene sequences were retrieved from 48 soil samples, with 42,769–61,239 sequences obtained from each sample (mean = 51,458). BS had the lowest bacterial diversity and richness compared with FS and SS (Table 2). The number of OTUs and the Chao and ACE indices decreased significantly in soils receiving N fertilizers compared with those in the N0 treatment in SS, whereas no significant difference was found among the four N treatments in FS and BS.

**Figure 2:**
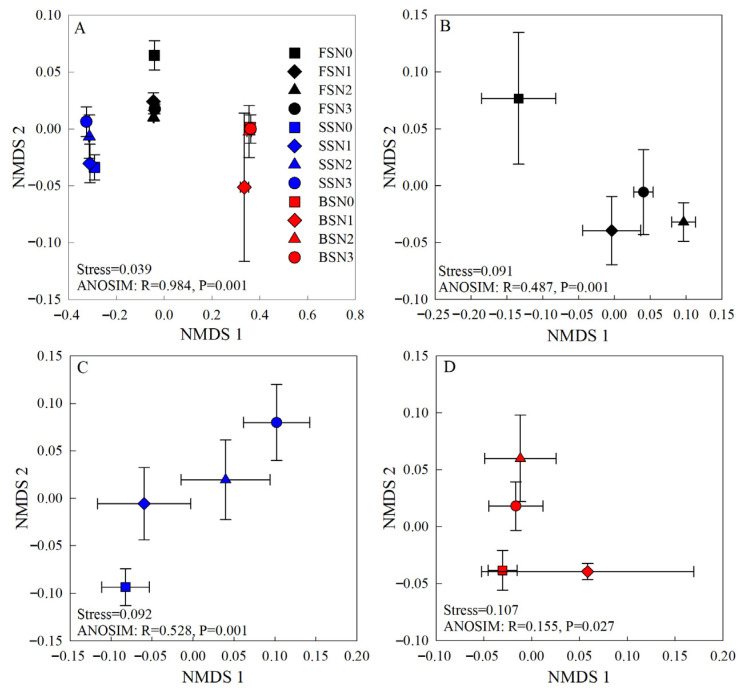
Non-metric multidimensional scaling ordination for bacterial communities from 48 soil samples for all soils (**A**), FS (**B**), SS (**C**), and BS (**D**) based on Bray–Curtis dissimilarities (mean + se, *n* = 4). Significant differences in sample clustering are measured by ANOSIM.

Bacterial community structures were visualized using NMDS based on Bray–Curtis distances calculated from the OTU table. This analysis revealed a clear separation among samples from different soils and showed that the bacterial community structure was significantly affected by soil type. In addition, the bacterial community was influenced by N fertilization to varying degrees across different soil types (Figure 2). The distribution of bacterial communities was not clearly separated by N-addition rates in BS. The ANOSIM results further confirmed that N fertilization significantly altered bacterial community structures across the three soil types; however, the effect was stronger in the FS and SS (*p* = 0.001) than in the BS (*p* = 0.027).

After a comprehensive analysis of the three soil types, we observed that the alpha diversity indices were negatively associated with soil moisture, OM, TN, AN, and AP, but significantly positively associated with soil pH across all samples (Table 3). In BS, Spearman’s correlation analysis indicated no significant relationship between bacterial alpha-diversity and soil characteristics (Table S3). For FS, the number of OTUs was significantly and negatively correlated with AP. In SS, OTUs, Chao, and ACE exhibited significant positive correlations with soil pH but significant negative correlations with NO_3_^−^-N.

### 3.3. Taxonomy of Soil Bacteria

**Figure 3:**
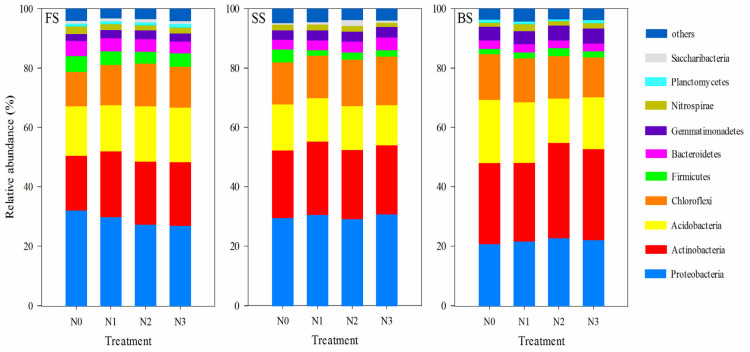
Relative abundance of dominant bacterial groups (phylum level) under different N treatments in three soil types. The groups accounting for 1% are shown, whereas those accounting for <1% are combined.

Generally, FS, SS, and BS soils had similar primary phyla under different N treatments. The dominant phyla were Proteobacteria, Actinobacteria, Acidobacteria, Chloroflexi, Firmicutes, Bacteroidetes, Gemmatimonadetes, Nitrospirae, Planctomycetes, and Saccharibacteria (>1%) (Figure 3). The relative abundances of the top five phyla (Proteobacteria, Actinobacteria, Acidobacteria, Chloroflexi, and Firmicutes) accounted for 84.2–85.8% and 85.4–86.4% in FS and SS, and the top five phyla (Actinobacteria, Proteobacteria, Acidobacteria, Chloroflexi, and Gemmatimonadetes) accounted for 87.9–89.4% in the BS. The impact of N fertilization on the relative abundance of bacterial phyla differed across various soil types. In the SS, the N application led to a marked decline in the relative abundance of Firmicutes but a significant increase in that of Saccharibacteria compared with the N0 treatment. However, in FS and BS, different N treatments did not cause marked differences in the relative abundance of the predominant phyla. Meanwhile, we also analyzed the effects of N application on the genus level (Figure S1). For FS soil, Bacillus was the dominant genus across all N treatments, and RB41, Streptomyces, and Sphingomonas also contributed to the community composition, but their relative abundances varied among treatments. In the SS soil, Sphingomonas was the most prominent genus, and its abundance increased with the increase in N application rate. Similar to FS, other genera like RB41, Streptomyces, and Nocardioides were present, but Thiobacillus occurs only in SS. Regarding BS soil, RB41 was the dominant genus; Gaiella, Streptomyces, and Rubrobacter showed distinct patterns of relative abundance changes with varying N levels. Overall, N application treatments influenced the relative abundance of bacterial genera in the three soil types, though the specific response patterns differed among soils, reflecting the heterogeneity in soil microbial community responses to N input.

**Figure 4:**
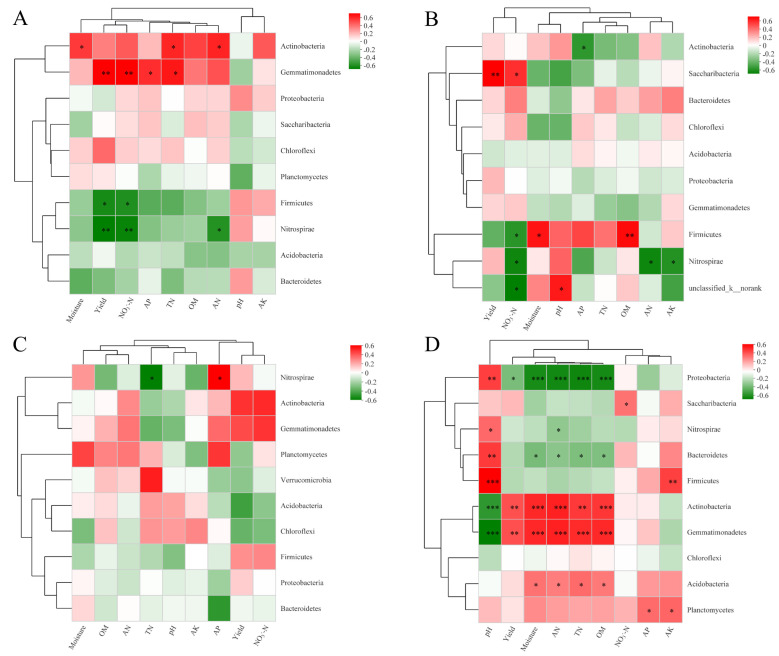
Pearson’s correlation heatmap between bacterial communities and soil properties in FS (**A**), SS (**B**), BS (**C**), and three soils (**D**). R-values are displayed in different colors, as indicated by the color code on the right of the heatmap. Significance levels are denoted by * *p* < 0.05, ** *p* < 0.01, and *** *p* < 0.001.

To explore the associations between predominant bacterial phyla and environmental variables in all samples, Pearson’s correlation analysis was carried out (Figure 4). In FS, NO_3_^−^-N and yield correlated marked positive relationship with the relative abundance of Gemmatimonadetes but correlated significantly negatively with the relative abundances of Firmicutes and Nitrospirae. In SS, NO_3_^−^-N and yield were significantly and negatively correlated with the relative abundance of Firmicutes. After analyzing the three soil types, we discovered that the relative abundances of Actinobacteria and Gemmatimonadetes had significant positive correlations with AN, TN, soil moisture, and OM, but these variables exhibited a significant negative correlation with soil pH. However, the Proteobacteria displayed the opposite relationship; they were significantly and negatively correlated with AN, TN, moisture, and OM, but significantly and positively correlated with soil pH. Additionally, soil pH was significantly and positively correlated with the relative abundances of Bacteroidetes and Firmicutes. The heatmaps illustrate the Pearson correlations between bacterial genera and environmental variables in FS, SS, and BS soils (Figure S2). In FS soil, *Ensifer* and *Microvirga* showed a significant positive correlation with pH. *Bacillus* and *Steroidobacter* exhibited a significant negative correlation with NO_3_^−^-N and yield. For SS, *Nitrospira* was significantly positively correlated with yield, *Sphingomonas* had negative correlations with pH, and these genera have the closest relationship with NO_3_^−^-N. In BS, *Kribbella* was positively correlated with NO_3_^−^-N, AN, and yield, while *RB41* had a significant negative correlation with yield. Overall, bacterial genera exhibited distinct correlation patterns with soil properties and yield across the three soils, indicating that soil type drives the associations between microbial communities and soil fertility or yield-related factors.

### 3.4. Bacterial Communities with Significant Differences

**Figure 5:**
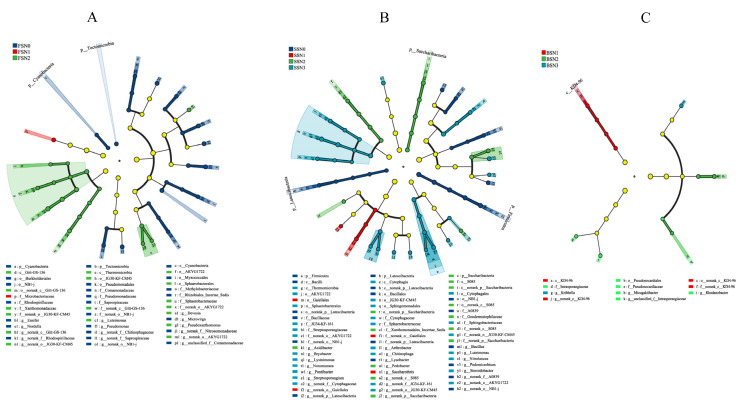
Cladogram showing the phylogenetic distribution of the bacterial lineages associated with soil from the four N treatments in FS (**A**), SS (**B**), and BS (**C**). The taxa with absolute LDA scores over 3 and *p*-values less than 0.05 are shown. Circles indicate phylogenetic levels from phylum to genus. The diameter of each circle is proportional to the abundance of the group.

In order to identify biomarkers in different treatments, we used the LEfSe score to analyze the microbial community data from phylum to genus level, and the groups with LDA scores > 3 were confirmed using this measure (Figure 5). It should be noted that the taxonomic resolution of LEfSe analysis is constrained by the targeted 16S rRNA gene region; for some taxa, classification could only be resolved to higher taxonomic ranks rather than species or strain level. This limitation means we cannot link these taxa to fine-scale functional differences and should be considered when interpreting their potential as indicator taxa. In FS, only the Microbacteriaceae family was significantly changed in the N1 treatment. In the N2 treatment, the AKYG1722 (from order to genus), JG30_KF_CM45 (from order to genus), Gitt_GS_136 (from class to genus), Sphaerobacteraceae (from order to family), Thermomicrobia (class), Nitrolancea, Devosia, Luteimonas, and Pseudoxanthomonas (genus) significantly changed (Figure 5A). In SS, Gaiellales (from order to genus) and the *Saccharothrix* genus were significantly changed in the N1 treatment. In the N2 treatment, Saccharibacteria (from phylum to genus), S085 (from class to genus), Xanthomonadales_Incertae_Sedis, Geodermatophilaceae (family), and Acidibacter (genus) were significantly changed. In the N3 treatment, AKYG1722 and JG30_KF_CM45 (from order to genus), Sphaerobacterales (order and family), Cytophagales (from class to genus), Thermomicrobia (class), Streptosporangiaceae (family), *Pontibacter*, *Chitinophaga*, *Arthrobacter*, and *Chitinophage* genera were significantly changed (Figure 5B). In BS, KD4_96 (from class to genus) was significantly changed in the N1 treatment. Pseudonocardiales (order and family), Intrasporangiaceae (family and genus), *Mizugakibacter*, and the *Rhodanobacter* genus were significantly changed in the N2 treatment, and only the *Kribbella* genus was significantly changed in the N3 treatment (Figure 5C).

As mentioned previously, we observed large variations in the bacterial communities from the phylum to genus levels under the four N treatments among the three soil types (Figure S3). In three soil types, the first three species of bacteria with the most significant differences were Thermomicrobia class, JG30_KF_CM45 order, and its family (FS); Firmicutes phylum, Bacilli class, and Bacillales order (SS); and KD4_96 class, Intrasporangiaceae family, and its genus (BS). These taxa may serve as potential indicators to reflect shifts in the soil bacterial community structure under N application conditions, though their functional relevance should be interpreted cautiously due to the taxonomic resolution limitations noted above.

### 3.5. The Abundance of N-Cycling-Related Genes

**Figure 6:**
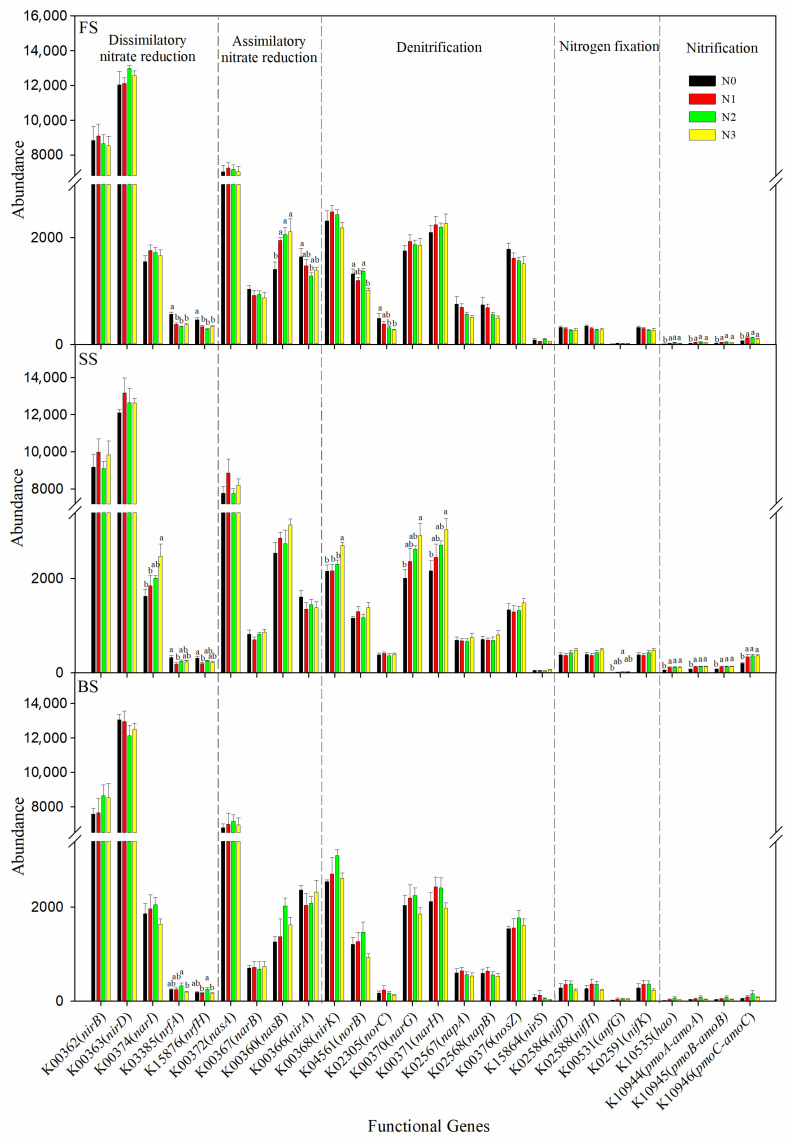
Abundance of selected orthologs associated with the nitrogen cycle at different N rates in the three soil types, derived from PICRUSt2 analysis. Bars with different letters are significantly different (*p* < 0.05).

In addition to microbial communities, soil N-cycling processes are also shaped by the N fertilizer and soil types. We analyzed the abundances of 26 bacterial genes implicated in the N metabolism, including dissimilatory nitrate reduction (DNRA), assimilatory nitrate reduction (ANRA), denitrification, N fixation, and nitrification. Absolute gene abundance values were normalized to the number of valid reads per sample to account for variations in sequencing depth among samples. Among these genes, DNRA genes were the most abundant across three soils, followed by ANRA, denitrification, N fixation, and nitrification genes. Overall, the abundance of genes related to N cycling differed significantly among the soil types, and the distribution of these genes displayed a leap trend. The SS had the highest abundance of *nirB*, *narI*, *nasA*, *nasB*, *narG*, *narH*, *napA*, *nifD*, *nifH*, *nifK*, and nitrification genes among the three soils (Figure S3). Investigations into the variation in N-cycle-associated gene abundance across different treatments at three sites showed that N fertilizer application significantly enhanced the abundance of some nitrification genes (*hao*, *pmoA-amoA*, *pmoB-amoB*, *pmoC-amoC*) in FS and SS, *nasB* in FS, and *narI*, *nirK*, *narG*, *narH*, and *anfG* in SS. Yet, N fertilizer significantly decreased the abundance of *nrfA* and *nrfH* in FS and SS. Specifically, most genes exhibited no significant change in relative abundance under different N treatments in BS, while the N2 treatment significantly increased the abundance of *nrfA* and *nrfH* compared with the N3 treatment (Figure 6). Table S4 presents Spearman’s correlation coefficients between N cycling genes and bacterial genera in FS, SS, and BS soils. The denitrification genes (*norC*, *narG, narH*, *napA*, and *napB*) were significantly negatively correlated with *RB41* in FS and SS. The *napA*, *napB,* and *nosZ* genes were significantly positively correlated with *Sphingomonas* in three soils; this indicates that *Sphingomonas* facilitates the denitrification process. In addition, the *nrfH* gene was significantly positively correlated with *Bacillus* in all three types of soils, indicating that *Bacillus* may promote the DNRA process.

### 3.6. Relationships Among Environmental Variables, Bacterial Communities, and N-Cycle-Related Genes

**Figure 7:**
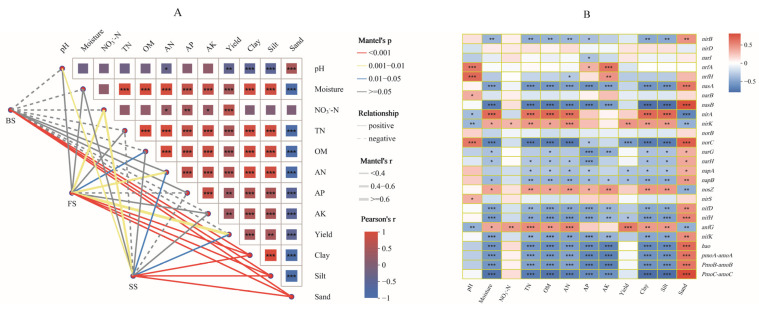
(**A**) The bacterial community composition of three soils based on Bray–Curtis distance is related to each edaphic factor by the Mantel test. The width of the line represents the partial Mantel’s r statistic, and the color of the line denotes the statistical significance based on 999 permutations. The pairwise correlation of environmental factors is shown by Pearson’s correlation coefficient with a color gradient, and straight and dashed lines represent positive and negative correlations, respectively. (**B**) Correlation between N-cycling function and edaphic factors and wheat yield. Colors represent Spearman correlations. Asterisks indicate the significance level: * *p* < 0.05, ** *p* < 0.01, *** *p* < 0.001.

Fertilization-induced changes in wheat yield of different soil types are closely associated with soil physicochemical properties and functional microbes. Mantel test revealed that soil properties were strongly correlated with bacterial communities. The soil texture had the most influence on determining the soil bacterial community (*p* < 0.001). The soil moisture, AN, TN, OM, AK, AP, and pH also made significant contributions across the three soil types. In FS, soil NO_3_^−^-N, AN, and OM contents and yield were significantly related to the bacterial community structure. For SS, the pH, NO_3_^−^-N, AN contents, and yield were important factors that correlated closely with bacterial community composition. However, in BS, none of the soil properties had a significant relationship with the bacterial community structure (Figure 7A). Pearson’s analysis between the N-cycle-related genes and soil properties showed that *nasA*, *nasB*, *nifD*, *nifH*, *nifK*, *hao*, *pmoA-amoA*, *pmoB-amoB*, and *pmoC-amoC* had significant negative relationships with soil moisture, TN, OM, AN, AP, AK, soil clay, and silt content, and yield, and NO_3_^−^-N were significantly positive with *nirK* and *anfG*, while *norC*, *napA*, *napB*, and *nifH* showed significant negative relationships with wheat yield. In addition, the relationships between sand content and the abundance of N-cycle-related genes were opposite to soil clay and silt content (Figure 7B).

### 3.7. The Effects of N Fertilization-Driven Soil and Bacteria Properties on Wheat Yield

**Figure 8:**
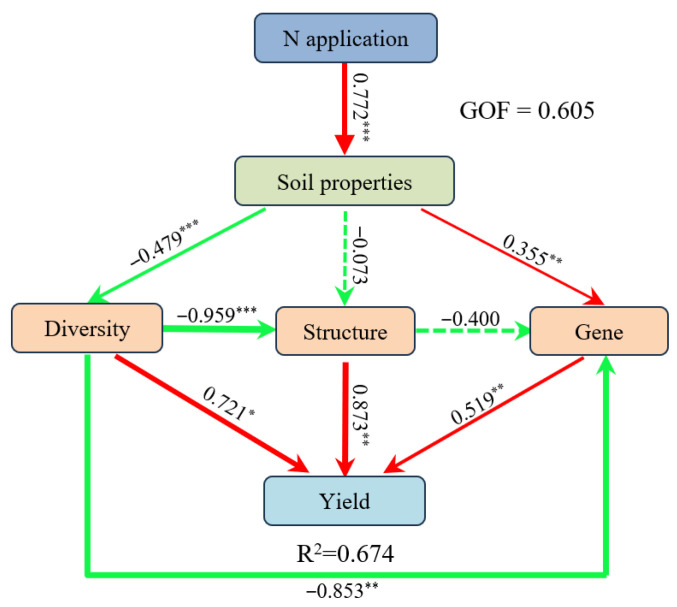
The partial least-squares path model (PLS-PM) was used in N fertilization treatments across three soil types. Soil characteristics included pH and NN, diversity included sobs and chao, genes included *nirK*, *nrfH,* and *anfG*. The red and green solid lines represent significantly positive and negative relationships, respectively. The dotted line indicates non-significant effect. The thickness of a line is proportional to the path coefficient. Asterisks indicate the significance level: * *p* < 0.05, ** *p* < 0.01, *** *p* < 0.001. R^2^ indicates the variance of dependent variable explained by independent variables.

PLS-PM (Figure 8) showed N application significantly altered soil properties (pH, NO_3_^−^-N), and these soil property changes were statistically linked to wheat yield via associated shifts in soil bacterial community diversity, structure, and functional genes. Specifically, N application exhibited a strong statistical association with soil characteristics (0.772, *p* < 0.001), with NO_3_^−^-N being the primary driver of this association. Soil properties negatively correlated with bacterial diversity (−0.479, *p* < 0.001) and positively associated with functional gene abundance (0.355, *p* < 0.01), with N fixation-related gene (*anfG*) being the most responsive functional gene groups. Bacterial diversity showed a marked negative correlative relationship with community structure (−0.959, *p* < 0.001). Structural factors significantly promoted yield (0.873, *p* < 0.01), whereas both diversity (0.721, *p* < 0.05) and genetic factors (0.519, *p* < 0.01) also exerted a positive effect on yield. The model explained 67.4% of yield variation and had a goodness-of-fit (GOF) of 0.605. These results highlight that N-induced soil changes are correlatively connected to yield through associated shifts in microbial traits.

## 4. Discussion

### 4.1. The Responses to N Fertilization of Wheat Yield Soil Physicochemical Properties

Recent studies have shown that increasing N input during the wheat season helps maintain optimal soil N levels [43], but application above 240 kg N ha^−1^ reduced N use efficiency [44]. In this study, the highest yield was obtained in the N2 treatment for three soils (Figure 1), and the N2 treatment had appropriate inorganic N content (Table 1), thereby improving plant N availability. Due to their unique initial characteristics [45], different soil types exhibited varied responses to N fertilization. In this study, the physiochemical properties of the three soils were significantly affected by N fertilization (Table 1). In agreement with previous studies, N treatments decreased pH regardless of soil type and acidified the soil [4]. In SS, we observed a relatively large change in pH (from 7.65 in N0 to 7.09 in N3) compared with that in FS (from 7.72 to 7.25) and BS (from 7.28 to 6.88). Soil pH is a major determinant of soil microbial community, and previous studies have shown that long-term N application causes significant acidification of soil via nitrification-mediated release of free H^+^ [46,47]. Yu et al. showed a similar result, in which the pH of sandy soil showed a more significant pH decrease with N fertilization compared to alluvial and clay soils because lowest buffer capacity in the sandy soil [48]. Such differences highlight that N-induced physicochemical changes are not universal but are modulated by soil-specific properties, challenging the notion of a one-size-fits-all response to N fertilization. Generally, N application can increase NO_3_^−^-N content by increasing potential nitrification rates and improving nitrate reductase [49], which can regulate the soil microbial community induced by plant growth under increased soil nutrients [50]. In FS, N treatment increased OM, TN, AN, and NO_3_^−^-N, which is consistent with the findings of an earlier investigation, suggesting that C and N availability may be an important factor in controlling bacterial diversity after N application in the fluvo-aquic soil [51,52]. Changes in soil physiochemical properties caused by N fertilization can directly affect crop production, and OM is an important underlying factor affecting crop yields by boosting microbial biomass [53].

### 4.2. The Responses of Soil Bacterial Diversity to N Fertilization

It was reported that while soil bacterial diversity remained unchanged, richness significantly increased with soil coarseness (quantified by sand percentage) [54]. The bacterial diversity of BS was low compared to FS and SS (Table 2), which aligns with previous findings showing that sand content positively correlates with diversity and richness, whereas clay shows an inverse relationship [55]. This phenomenon can be attributed to the higher connectivity in clay soils, which may lead to enhanced spatial linkages and niche homogeneity, thereby fostering microbial interactions while limiting community diversification and maintaining microbial richness and diversity [12]. However, this texture-diversity relationship is not universal; previous studies have shown that N fertilization induced notable alterations in the structure and diversity of bacterial communities, particularly leading to a reduction in bacterial diversity within sandy loam soil [56], silt loam [1], and brown soil [57]. In the present study, N fertilization caused significant decreases in the OTUs, and Chao and ACE indices in SS, but not in the FS and BS. This might be attributed to the changes in bacterial diversity within agricultural soils, which are influenced by physicochemical factors such as soil texture [54], aeration conditions [9], N content [58], and soil pH [59]. Wang et al. also reported that the bacterial response to N fertilization could be highly dependent on the background nutrient availability or acidification status [60]. The soil microbial composition and diversity correlate closely with soil properties [61,62]. Correlation analysis indicated a significant negative relationship between bacterial diversity and richness and the concentrations of NH_4_^+^, NO_3_^−^-N, TN, and OM. In the present study, the OTUs, Chao, and ACE indices exhibited a positive correlation with pH, whereas they showed a negative correlation with NO_3_^−^-N in SS. Only the AP showed a significant negative relationship with the OTUs in the FS. These differences confirm that the drivers of microbial diversity under N input are not universal but depend on which soil properties are most limiting at a given site.

The NMDS analyses showed a significant change in the β-diversity of soil bacteria under N application, and we observed a clear distinction among different soil types and N treatments; this finding aligns with those from earlier research [63,64]. The ANOSIM results revealed that the influences increased following the order of BS < FS < SS (Figure 2). Previous studies have reported that pH and soil N availability are associated with changes in soil bacterial community structure [49,65]. In the current study, the results of the Mantel test showed that soil AN had the strongest influence on shaping the soil bacterial community structure in all samples (Figure 7A). Moreover, TN, OM, AK, AP, and pH also contributed significantly across the three soil types. In FS and SS, the contents of NO_3_^−^-N and AN were related significantly to the structure of the bacterial community, which is consistent with previous studies [66]. Overall, the structure of the bacterial community correlated closely with the soil physicochemical properties and was affected by N fertilization.

### 4.3. The Responses of Soil Bacteria Taxa to N Fertilization

High-throughput sequencing demonstrated that across the three soil types, the predominant bacterial phyla were Proteobacteria, Actinobacteria, Acidobacteria, Chloroflexi, and Firmicutes, which accounted for an average of 86.5% of the total bacterial sequences obtained in this study; this is consistent with findings from prior research conducted on agricultural soils [67]. However, the response of these phyla to N enrichment is far from universal in the literature. For example, Proteobacteria (encompassing many denitrifiers) and Actinobacteria are often classified as copiotrophic groups that increase with long-term N input in agricultural fields [13]. Yet, other studies report their significant decrease under N addition in arid deserts [68]. In addition, members of the Actinobacteria can perform many functions in soils, such as the decomposition of organic material and nitrogen fixation [69]. In our study, the abundance of Actinobacteria increased under N fertilization in all soils (Figure 3), aligning with the copiotrophic hypothesis, but Proteobacteria showed soil-specific variation, decreasing in FS but increasing in BS. These findings indicate that N addition-induced shifts in the abundance of copiotrophic bacteria were not uniform, implying the adaptive mechanisms of bacterial community composition to N fertilizer vary according to the cropping systems and soil properties. In general, Acidobacteria are considered oligotrophic taxa, thriving in nutrient-poor conditions and contributing to recalcitrant carbon degradation [70]. Our observation of the highest abundance of Acidobacteria under N2 treatment in FS aligns with one study reporting that Acidobacteria increase under high N [71]. While N fertilization elicited consistent phylum-level responses across the three soil types, the same treatments triggered divergent microbial reactions under distinct soil conditions, likely due to functional redundancy within these phyla.

Soil bacterial communities can be affected directly by N fertilization, and indirectly by changing soil chemical properties [72]. Soil pH is regarded as a major driver in changing soil microbial communities [73]. Pearson correlation analysis showed that soil pH was significantly positively correlated with Proteobacteria, Bacteroidetes, and Firmicutes, but was significantly negatively correlated with Actinobacteria and Gemmatimonadetes across three soil types (Figure 4D). Our previous results found that OM, AP, and AK were positively correlated with the relative abundance of Actinobacteria, whereas these bacteria correlated negatively with AN under irrigated and N application conditions [74]. In FS and SS, the relative abundance of Firmicutes correlated significantly and negatively with yield (Figure 4A,B), which was consistent with the results of a previous study [48]. In all samples, we observed that Actinobacteria and Gemmatimonadetes correlated positively with soil moisture, AN, TN, OM, and yield (Figure 4D). A previous study also found that the relative abundance of Gemmatimonadetes correlated significantly and positively with plant biomass [75]. Wang et al. reported that soil chemical properties were not obviously related to the Bacteroidetes, Planctomycetes, and Chloroflexi in red clay soil in southern China [76]. Our results revealed that Chloroflexi was also not significantly correlated with soil properties; however, Bacteroidetes correlated positively with pH (*p* < 0.001) and negatively with AN and TN. These inconsistencies confirm that taxon-environment-yield relationships are not universal but are modulated by soil-specific conditions, and ignoring these nuances oversimplifies agroecosystem dynamics.

Based on LEfSe analysis (Figure 5), there were 43, 62, and 11 bacterial species that changed significantly in the FS, SS, and BS, respectively. The Thermomicrobia class, *Nitrolancea* genus, AKYG1722 (from order to genus), and JG30_KF_CM45 (from order to genus) were significantly enriched under N treatments in FS and SS soils. Zhong et al. reported that the Microbacteriaceae family and the AKYG1722 order were significantly increased under N treatments, which is consistent with our results in the FS [52]. Burkholderiales (from order to genus), Pseudomonadaceae (from order to genus), and the Myxococcales order were significantly enriched under the N0 treatment in the FS. A previous study also found that the Burkholderiales_incertae_sedis and Pseudomonadaceae families were enriched under the N0 treatment; however, the Myxococcales order was enriched under low N treatment (15 kg ha^−1^) [77]. In addition, we found that Chitinophaga and Steroidobacter were enriched under the N3 treatment, and *Lysobacter* (genus) was enriched under the N0 treatment in the SS. However, Zhang et al. stated that *Lysobacter* (genus) was enriched under high N treatment (200 kg ha^−1^) [77]. Bacillales (order), Firmicutes (phylum), and Bacilli (class) were biomarkers of SS. Our study found that the Thermomicrobia class was one of the biomarkers for both FS and SS, which is consistent with the result of Li et al., who reported that Thermomicrobia was the biomarker in a fish farm via LEfSe analysis [78]. This indicates that the Thermomicrobia class is frequently identified as a biomarker across different ecosystems. In BS, unclassified *Intrasporangiaceae* was the species with the largest change in abundance under N fertilizer treatment and served as an indicator of paddy soil in previous studies [79]. Thus, different soils appeared to cause no consistent shifts of bacterial communities under the N conditions applied in the present study, although we revealed similar effects on bacterial communities induced by N fertilization in two soil types. This further confirms that taxon-environment relationships are not universal but are modulated by soil-specific conditions.

### 4.4. Bacterial N-Cycling Genes and the Relationships with Environmental Factors

Elucidating the soil N-cycling process is significant for improving N use, decreasing N loss, and further increasing crop yield. Long-term inorganic N fertilization led to high levels of nitrate and ammoniacal N, encouraging the proliferation of denitrifying bacteria and causing N loss in soil [80,81]. Various nitrogen cycle processes are mediated by specific functional genes that are crucial for their respective functions. These include *nifH* for nitrogen fixation, *amoA*/*B* for nitrification, *narG*, *napA*, *nirK*/*S*, and *nosZ* for denitrification, as well as *nirB*/*D* and *nrfA*/*H* for dissimilatory nitrate reduction [82]. In general, N addition had a negative [26] or no significant influence [83] on the abundance of the nitrification genes. However, our results showed that N fertilizers significantly increased the abundance of some nitrification genes (*hao*, *pmoA-amoA*, *pmoB-amoB*, *pmoC-amoC*) in FS and SS. Similar results were noted by Liu et al. [84], who discovered a notable increase in the abundance of the *hao* gene following the application of urea and demonstrated a significantly stronger association with the level of nitrogen present in the soil, probably due to higher soil N concentrations, which alleviates the competition for nutrients between autotrophic and heterotrophic nitrifying microbes [85]. This can partially explain the reason of soil NO_3_^−^-N content significantly increased in N applied treatments. Denitrification, a microbially facilitated process, reduces nitrate and nitrite to nitric oxide (NO), nitrous oxide (N_2_O), and nitrogen (N_2_), playing a crucial role in the transformation of the nitrogen cycle. N fertilizer significantly increased the abundance of denitrification encoding genes (*nirK*, *narG*, *narH*) in SS. In line with previous studies in semiarid regions, which observed the abundance of *narG*, *nirK*, *nirS,* and *nosZ* genes increased significantly following N application rates [83], likely because of the increased N availability due to N addition, provided considerable NO_3_^−^-N as the substrate of denitrification [86]. The present study further supported previous studies that N fertilization increases the abundance of N functional genes [87], particularly evident is the nitrification and denitrification [88,89]. It should be noted that PICRUSt2 predictions rely on the completeness and representativeness of the reference database and cannot capture functional traits of uncultured or poorly represented taxa in the database. To validate and refine these functional inferences, complementary approaches, such as shotgun metagenomics or metatranscriptomics, would be valuable in future studies.

In the current study, the abundances of genes related to N fixation (except *anfG*) significantly negatively correlated with soil TN, OM and AN, which is partially consistent with a previous study that the abundance of N fixation genes was significantly negatively correlated with soil TN content [83], a meta-analysis also revealed that N fertilizer suppresses N fixation potential [90]. However, Li et al. observed that the relative abundance of *nifH* had a positive correlation with the contents of OM, TN, NO_3_^−^, and NH_4_^+^ [31]. This is because N-cycling processes are influenced by various physicochemical parameters under different soil conditions. A previous study revealed that soil pH has some influence on the abundance of denitrification genes, and relevant genes such as *nirK*, *norC*, and *norB* were positively correlated with pH [91]. Our study found that pH was significantly positively correlated with *nirS* and *norC* genes, but negatively correlated with *nirK* genes. It has been reported that soil N-cycling genes (*narG*, *nirK*, *nosZ*, *amoA*, *amoB*, and *nifH*) are significantly negatively correlated with *RB1*, but there is no significant association between genes and *Sphingomonas* in the apple orchard [92]. By analyzing the relationship between functional genes and bacterial genera, we found that *nirA*, *nirK*, and *narG* were significantly negatively correlated with *RB1* in FS and SS, and *nirK* and *norB* were significantly positively correlated with *Sphingomonas* across three soils. This might result from differences in cultivated plants and soil properties. The analysis of the relationship between functional gene abundance, soil properties, and bacterial communities revealed that environmental conditions significantly influence soil N-cycling processes.

The sustainability of crop yields and soil productivity is significantly influenced by modifications in soil physicochemical properties, including soil texture, nutrient availability, organic matter, and microbial traits [93]. In the present study, PLS-PM analysis revealed that N fertilizer was significantly associated with changes in soil properties, which were in turn statistically linked to shifts in bacterial communities, and the combined effects of these associations were correlated with variations in wheat yield (Figure 8). For example, soil pH and NO_3_^−^-N were key drivers controlling wheat yield, through mediation of bacterial diversity (sobs and chao), structure, and N functional genes (*nirK*, *nrfH*, and *anfG*). Liu et al. [94] and Li et al. [95] also reported that soil microorganisms exhibited both direct and indirect statistical associations with crop yield by modifying soil properties, demonstrating that intercropping improves system productivity by modifying soil bacterial communities using PLS-PM analysis. These results highlight that N-induced soil changes are correlatively linked to yield through associated shifts in microbial traits. The identified statistical linkages provide potential targets for optimizing N management, but they should be interpreted within the context of agroecosystem complexity. Future research should focus on elucidating the functional roles of the identified indicator species and developing targeted microbiome manipulation approaches, such as field validation of microbial inoculants or nutrient-microbe co-regulation to test whether the observed correlative links between microbes and yield reflect underlying causal relationships, thereby improving N use efficiency and mitigating the environmental impacts associated with N fertilization.

## 5. Conclusions

This study provides a comprehensive evaluation of the effects of N fertilization on wheat yield, soil properties, and soil bacterial communities across three soil types. Our findings indicate that the optimal N application rate (240 kg N ha^−1^) resulted in maximum wheat yield, and yields differed significantly across soil types, with SS having the lowest yield. N fertilization significantly altered soil physicochemical properties, with notable impacts on pH and NO_3_^−^-N content, which in turn drove shifts in bacterial diversity and community structure. Abundances of nitrification and denitrification genes significantly increased in SS, while changes were minor in BS. Specific bacterial taxa and N-cycling genes exhibited distinct responses to N fertilization, which were strongly modulated by soil type. The Thermomicrobia class, Bacillales order, and unclassified family Intrasporangiaceae are the indicator taxa for FS, SS, and BS, respectively. Furthermore, Partial Least Squares Path Modeling (PLS-PM) revealed that N fertilization-induced changes in soil properties were indirectly associated with yield variation via their statistical links to bacterial communities. This study provides valuable insights for optimizing N management strategies to enhance wheat productivity while promoting soil health and sustainability in agricultural systems with similar soil types.

## Figures and Tables

**Table 1:** Effects of N fertilization regimes on soil properties in three soil styles.

Soil	N Rate	Moisture (%)	pH	OM (g kg^−1^)	TN (g kg^−1^)	AN (mg kg^−1^)	AP (mg kg^−1^)	AK (mg kg^−1^)	NO_3_^−^-N (g kg^−1^)
FS	N0	19.11 c	7.72 a	17.43 c	0.88 b	97.98 b	19.93 b	362.58 b	15.98 c
	N1	21.10 a	7.74 a	20.77 a	1.06 a	114.31 a	21.39 b	392.42 a	44.82 a
	N2	20.13 b	7.44 b	19.34 b	1.08 a	112.20 a	23.98 a	359.93 b	41.68 a
	N3	20.36 b	7.25 c	19.28 b	1.05 a	111.68 a	16.85 c	407.72 a	31.88 b
ANOVA *p*-values		<0.001	<0.001	0.288	0.245	0.006	0.154	0.312	<0.001
SS	N0	12.24 a	7.65 a	8.02 a	0.48 a	76.38 ab	14.51 a	152.04 b	11.27 c
	N1	9.98 b	7.32 b	6.59 c	0.42 b	73.74 b	9.98 c	95.81 c	21.17 b
	N2	9.42 b	7.22 b	7.52 ab	0.49 a	74.27 b	12.16 b	151.41 b	26.78 b
	N3	9.78 b	7.09 c	6.92 bc	0.47 a	83.23 a	15.06 a	191.43 a	36.56 a
ANOVA *p*-values		<0.001	<0.001	0.001	0.001	0.063	<0.001	<0.001	<0.001
BS	N0	25.30 c	7.28 a	27.16 a	1.42 a	132.74 b	19.75 b	360.95 a	18.70 c
	N1	26.77 b	7.17 a	22.79 c	1.29 b	126.43 c	18.79 b	333.33 c	25.63 b
	N2	23.40 d	7.02 b	24.01 b	1.36 ab	135.38 b	19.15 b	347.59 b	28.60 b
	N3	28.31 a	6.88 c	25.27 b	1.32 b	143.81 a	22.13 a	287.34 d	41.71 a
ANOVA *p*-values		<0.001	<0.001	<0.001	0.021	<0.001	0.035	<0.001	<0.001
Soil styles (*p*-values)		**<0.001**	**<0.001**	**<0.001**	**<0.001**	**<0.001**	**<0.001**	**<0.001**	**0.041**

OM: organic matter; TN: total nitrogen; AN: available nitrogen; AP: available phosphorus; AK: available. Values are means in four replicates with different letters in a column for the same soil type indicate significant difference among N treatments (*p* < 0.05), and bold *p*-values indicate significant difference within soil types.

**Table 2:** Estimators of bacterial diversity and richness fractions under different N-application treatments in three soil types.

Soils	N Rates	OTUs ^a^	Shannon	Chao	ACE
FS	N0	3207 ± 67 a	6.96 ± 0.05 a	4160 ± 93 a	4160 ± 93 a
	N1	3263 ± 7 a	6.98 ± 0.01 a	4274 ± 65 a	4293 ± 44 a
	N2	3200 ± 24 a	6.95 ± 0.01 a	4190 ± 43 a	4202 ± 24 a
	N3	3274 ± 36 a	6.98 ± 0.02 a	4222 ± 62 a	4240 ± 58 a
SS	N0	3487 ± 25 a	6.99 ± 0.04 a	4529 ± 19 a	4571 ± 32 a
	N1	3293 ± 33 b	6.81 ± 0.14 a	4339 ± 40 b	4303 ± 20 b
	N2	3293 ± 27 b	6.95 ± 0.05 a	4250 ± 54 b	4256 ± 38 b
	N3	3160 ± 61 c	6.88 ± 0.04 a	4080 ± 69 c	4079 ± 66 c
BS	N0	2217 ± 35 a	6.39 ± 0.03 a	2941 ± 22 a	2871 ± 31 a
	N1	2322 ± 60 a	6.47 ± 0.07 a	2995 ± 70 a	3003 ± 67 a
	N2	2283 ± 74 a	6.46 ± 0.07 a	2982 ± 55 a	2960 ± 65 a
	N3	2266 ± 57 a	6.40 ± 0.06 a	2948 ± 112 a	2930 ± 89 a

Data are the means ± standard errors, and different letters indicate significant differences among different N treatments. ^a^ OTUs: operational taxonomic units (97% similarity); sandy soils (SS), fluvo-aquic soil (FS), black soil (BS).

**Table 3:** Significant Spearman’s rank correlation coefficients between diversity indices and soil properties for all samples (*n* = 48).

Diversity Index	Moisture (%)	pH (g kg^−1^)	OM (g kg^−1^)	TN (mg kg^−1^)	AN (mg kg^−1^)	AP (mg kg^−1^)	AK (mg kg^−1^)	NO_3_^−^-N (g kg^−1^)
OTUs	−0.717 ***	0.493 ***	−0.748 ***	−0.768 ***	−0.793 ***	−0.478 **	−0.230	−0.185
Shannon	−0.549 ***	0.583 ***	−0.543 ***	−0.539 ***	−0.614 ***	−0.188	0.099	−0.024
Chao	−0.696 ***	0.566 ***	−0.715 ***	−0.738 ***	−0.787 ***	−0.416 **	−0.239	−0.147
Ace	−0.705 ***	0.560 ***	−0.712 ***	−0.722 ***	−0.774 ***	−0.421 **	−0.223	−0.160

OTUs: operational taxonomic units; OM: organic matter; TN: total nitrogen; AN: available nitrogen; AP: available phosphorus; AK: available. Significant levels: ** *p* < 0.01 and *** *p* < 0.001.

## Data Availability

The original contributions presented in this study are included in the article and Supplementary Materials. Further inquiries can be directed to the corresponding author.

## Supplementary Materials

The following supporting information can be downloaded at: https://www.mdpi.com/article/10.3390/microorganisms13102382/s1, Figure S1. Relative abundance of top 20 bacterial groups (genus level) under different N treatments in three soil types; Figure S2. Pearson correlation heatmap between bacterial genera and soil properties in FS, SS, and BS. R-values are displayed in different colors, as indicated by the color code on the right of the heatmap. Significance levels are denoted by * *p* < 0.05, ** *p* < 0.01, and *** *p* < 0.001; Figure S3: Indicator bacteria with LDA scores of 3 or greater in bacterial communities from the four N treatments in FS (A), SS (B), BS (C); Figure S4: Abundance of selected orthologs associated with nitrogen cycle in FS, SS, and BS, derived from PICRUSt2 analysis. Bars with different letters are significantly different (*p* < 0.05); Table S1: Basic soil properties from three soil styles of experimental sites at the start of the field experiment; Table S2. Effects of N fertilization regimes on soil properties in three soil styles; Table S3: Spearman’s rank correlation coefficients between diversity indices and soil properties for three soils (*n* = 16); Table S4. Spearman’s correlation coefficients between diversity indices and soil properties for three soils.

## References

[1] Ma Q., Qian Y.S., Yu Q.Q., Cao Y.F., Tao R.R., Zhu M., Ding J.F., Li C.Y., Guo W.S., Zhu X.K. (2023). Controlled-release nitrogen fertilizer application mitigated N losses and modified microbial community while improving wheat yield and N use efficiency. Agric. Ecosyst. Environ..

[2] Galloway J.N., Townsend A.R., Erisman J.W., Bekunda M., Cai Z., Freney J.R., Martinelli L.A., Seitzinger S.P., Sutton M.A. (2008). Transformation of the nitrogen cycle: Recent trends, questions, and potential solutions. Science.

[3] Guo Y.X., Chen Y.F., Searchinger T.D., Zhou M., Pan D., Yang J.N., Wu L., Cui Z.L., Zhang W.F., Zhang F.S. (2020). Air quality, nitrogen use efficiency and food security in China are improved by cost-effective agricultural nitrogen management. Nat. Food.

[4] Guo J.H., Liu X.J., Zhang Y., Shen J.L., Han W.X., Zhang W.F., Christie P., Goulding K.W.T., Vitousek P.M., Zhang F.S. (2010). Significant Acidification in Major Chinese Croplands. Science.

[5] Ghafoor I., Habib-ur-Rahman M., Ali M., Afzal M., Ahmed W., Gaiser T., Ghaffar A. (2021). Slow-release nitrogen fertilizers enhance growth, yield, NUE in wheat crop and reduce nitrogen losses under an arid environment. Environ. Sci. Pollut. Res..

[6] Xu A.X., Khan K.S., Wei X.X., Chen Y.F., Zhou Y.X., Sun C.R., Effah Z., Li L.L. (2025). Fertilizer nitrogen use efficiency and its fate in the spring wheat-soil system under varying N-fertilizer rates: A two-year field study using ^15^N tracer. Soil Till. Res..

[7] Jiang S.Q., Yu Y.N., Gao R.W., Wang H., Zhang J., Li R., Long X.H., Shen Q.R., Chen W., Cai F. (2019). High-throughput absolute quantification sequencing reveals the effect of different fertilizer applications on bacterial community in a tomato cultivated coastal saline soil. Sci. Total Environ..

[8] Hartmann M., Frey B., Mayer J., Mäder P., Widmer F. (2015). Distinct soil microbial diversity under long-term organic and conventional farming. ISME J..

[9] Berg G., Smalla K. (2009). Plant species and soil type cooperatively shape the structure and function of microbial communities in the rhizosphere. FEMS Microbiol. Ecol..

[10] Yin R.X., Li L.L., Li X., Liu H.F., Yao J.M., Ma C.Y., Pu L.L., Peng Y.T., Lei Z.W. (2025). Positive effects of nitrogen fertilization on the flavor ingredients of tea (Wuniuzao), soil physicochemical properties, and microbial communities. Environ. Technol. Innov..

[11] Chen Y., Jiang Z.Q., Ou J.M., Liu F.D., Cai G.Y., Tan K.M., Wang X.L. (2024). Nitrogen substitution practice improves soil quality of red soil (Ultisols) in South China by affecting soil properties and microbial community composition. Soil Till. Res..

[12] Obayomi O., Seyoum M.M., Ghazaryan L., Tebbe C.C., Murase J., Bernstein N., Gillor O. (2021). Soil texture and properties rather than irrigation water type shape the diversity and composition of soil microbial communities. Appl. Soil Ecol..

[13] Fierer N., Lauber C.L., Ramirez K.S., Zaneveld J., Bradford M.A., Knight R. (2012). Comparative metagenomic, phylogenetic and physiological analyses of soil microbial communities across nitrogen gradients. ISME J..

[14] Castellano-Hinojosa A., Strauss S.L., González-López J., Bedmar E.J. (2021). Changes in the diversity and predicted functional composition of the bulk and rhizosphere soil bacterial microbiomes of tomato and common bean after inorganic N-fertilization. Rhizosphere.

[15] Liang R., Hou R., Li J., Lyu Y., Hang S., Gong H., Ouyang Z. (2020). Effects of different fertilizers on rhizosphere bacterial communities of winter wheat in the North China Plain. Agronomy.

[16] Semenov M.V., Krasnov G.S., Semenov V.M., van Bruggen A.H.C. (2020). Long-term fertilization rather than plant species shapes rhizosphere and bulk soil prokaryotic communities in agroecosystems. Appl. Soil Ecol..

[17] Yao R.J., Yang J.S., Wang X.P., Xie W.P., Zheng F., Li H.Q., Tang H., Zhu C. (2021). Response of soil characteristics and bacterial communities to nitrogen fertilization gradients in a coastal salt-affected agroecosystem. Land Degrad. Dev..

[18] Yang G., Ma Y., Xu W., Ma X., Lu C. (2024). Spent mushroom substrate as a substitute for chemical fertilizer changes N-cycling genes and reduces N_2_O emission in different textured soils. Biol. Fertil. Soils.

[19] Zhang Y., Hu T., Wang H., Jin H., Liu Q., Lin Z., Liu B., Liu H., Chen Z., Lin X. (2021). How do different nitrogen application levels and irrigation practices impact biological nitrogen fixation and its distribution in paddy system?. Plant Soil.

[20] Ying D., Chen X.L., Hou J.F., Zhao F.C., Li P. (2023). Soil properties and microbial functional attributes drive the response of soil multifunctionality to long-term fertilization management. Appl. Soil Ecol..

[21] Tiong J., Sharma N., Sampath R., MacKenzie N., Watanabe S., Metot C., Lu Z., Skinner W., Lu Y., Kridl J. (2021). Improving nitrogen use efficiency through overexpression of alanine aminotransferase in rice, wheat, and barley. Front. Plant Sci..

[22] Li R.C., Gao Y.X., Chen Q., Li Z.L., Gao F., Meng Q.M., Li T.G., Liu A.R., Wang Q., Wu L. (2021). Blended controlled-release nitrogen fertilizer with straw returning improved soil nitrogen availability, soil microbial community, and root morphology of wheat. Soil Till. Res..

[23] Wang L.F., Wu K.K., Xiao F.R., Gong P., Xue Y., Song Y.C., Wang R.Z., Wu Z.J., Zhang L.L. (2025). Effect of biological denitrification inhibitor on N_2_O emissions from paddy soil and microbial mechanisms. Microorganisms.

[24] Wakelin S.A., Colloff M.J., Harvey P.R., Marschner P., Gregg A.L., Rogers S.L. (2007). The effects of stubble retention and nitrogen application on soil microbial community structure and functional gene abundance under irrigated maize. FEMS Microbiol. Ecol..

[25] Chen Z., Hou H.J., Zheng Y., Qin H.L., Zhu Y.J., Wu J.S., Wei W.X. (2012). Influence of fertilisation regimes on a nosZ-containing denitrifying community in a rice paddy soil. J. Sci. Food Agric..

[26] Shang S.S., Song M.H., Wang C.M., Dou X.M., Wang J.X., Liu F.F., Zhu C.Y., Wang S.Q. (2023). Decrease of nitrogen cycle gene abundance and promotion of soil microbial-N saturation restrain increases in N_2_O emissions in a temperate forest with long-term nitrogen addition. Chemosphere.

[27] Carey C.J., Dove N.C., Beman J.M., Hart S.C., Aronson E.L. (2016). Meta-analysis reveals ammonia-oxidizing bacteria respond more strongly to nitrogen addition than ammonia-oxidizing archaea. Soil Biol. Biochem..

[28] Müller C., Zhang L., Zipfel S., Topitsch A., Lutz M., Eckert J., Prasser B., Chami M., Lü W., Du J. (2022). Molecular interplay of an assembly machinery for nitrous oxide reductase. Nature.

[29] You L.C., Ros G.H., Chen Y.L., Yang X., Cui Z.L., Liu X.J., Jiang R.F., Zhang F.S., De Vries W. (2022). Global meta-analysis of terrestrial nitrous oxide emissions and associated functional genes under nitrogen addition. Soil Biol. Biochem..

[30] Pan H., Qin Y., Wang Y., Liu S., Yu B., Song Y., Wang X., Zhu G. (2020). Dissimilatory nitrate/nitrite reduction to ammonium (DNRA) pathway dominates nitrate reduction processes in rhizosphere and non-rhizosphere of four fertilized farmland soil. Environ Res.

[31] Li Q., Zhang Y., Hu J., Dai Q. (2022). Response of bacterial communities and nitrogen-cycling genes in newly reclaimed mudflat paddy soils to nitrogen fertilizer gradients. Environ. Sci. Pollut. Res..

[32] Zhang J., Zhao Z., Zhu C., Wang E., Brunel B., Li S., Zheng Q., Feng Z., Zhang H. (2025). Diverse peanut bradyrhizobial communities in Chinese soils: Insights from Eastern, Central, and Northern Henan Province. Microb. Ecol..

[33] Lu R.K. (2000). Methods of Soil and Agro-Chemistry.

[34] Mori H., Maruyama F., Kato H., Toyoda A., Dozono A., Ohtsubo Y., Nagata Y., Fujiyama A., Tsuda M., Kurokawa K. (2014). Design and experimental application of a novel non-degenerate universal primer set that amplifies prokaryotic 16S rRNA genes with a low possibility to amplify eukaryotic rRNA genes. DNA Res..

[35] Xu N., Tan G.C., Wang H.Y., Gai X.P. (2016). Effect of biochar additions to soil on nitrogen leaching, microbial biomass and bacterial community structure. Eur. J. Soil Biol..

[36] Campbell B.J., Polson S.W., Hanson T.E., Mack M.C., Schuur E.A.G. (2010). The effect of nutrient deposition on bacterial communities in Arctic tundra soil. Environ. Microbiol..

[37] Edgar R.C. (2010). Search and clustering orders of magnitude faster than BLAST. Bioinformatics.

[38] Pruesse E., Quast C., Knittel K., Fuchs B.M., Ludwig W., Peplies J., Glockner F.O. (2007). SILVA: A comprehensive online resource for quality checked and aligned ribosomal RNA sequence data compatible with ARB. Nucleic Acids Res..

[39] Sun S., Jones R.B., Fodor A.A. (2020). Inference-based accuracy of metagenome prediction tools varies across sample types and functional categories. Microbiome.

[40] Langille M.G.I., Zaneveld J., Caporaso J.G., McDonald D., Knights D., Reyes J.A., Clemente J.C., Burkepile D.E., Vega Thurber R.L., Knight R. (2013). Predictive functional profiling of microbial communities using 16S rRNA marker gene sequences. Nat. Biotechnol..

[41] Gao Y., Song X., Zheng W., Wu L., Chen Q., Yu X., Li Z., Li R., Gao F., Tian H. (2022). The controlled-release nitrogen fertilizer driving the symbiosis of microbial communities to improve wheat productivity and soil fertility. Field Crops Res..

[42] Segata N., Izard J., Waldron L., Gevers D., Miropolsky L., Garrett W.S., Huttenhower C. (2011). Metagenomic biomarker discovery and explanation. Genome Biol..

[43] Wang Y., Xu Y., Guo Q., Zhang P., Cai T., Jia Z. (2023). Adopting nitrogen deep placement based on different simulated precipitation year types enhances wheat yield and resource utilization by promoting photosynthesis capacity. Field Crops Res..

[44] Xiao X.C., Kang J., Li H.W., Liu Y., Yao C.S., Zhang Z., Liu Y., Sun W., Kang G.Z., Wang Z.M. (2023). Facilitating winter wheat sustainable intensification: Effects of two limited carbon-emission cultivation patterns in China’s Huang-Huai-Hai Region. Agric. Ecosyst. Environ..

[45] Zeglin L.H., Stursova M., Sinsabaugh R.L., Collins S.L. (2007). Microbial responses to nitrogen addition in three contrasting grassland ecosystems. Oecologia.

[46] Feng Y.Z., Grogan P., Caporaso J.G., Zhang H.Y., Lin X.G., Knight R., Chu H.Y. (2014). pH is a good predictor of the distribution of anoxygenic purple phototrophic bacteria in Arctic soils. Soil Biol. Biochem..

[47] Wang C.Y., Xiao H.G., Liu J., Zhou J.W., Du D.L. (2016). Insights into the effects of simulated nitrogen deposition on leaf functional traits of rhus typhina. Pol. J. Environ. Stud..

[48] Yu H.L., Ling N., Wang T.T., Zhu C., Wang Y., Wang S.J., Gao Q. (2019). Responses of soil biological traits and bacterial communities to nitrogen fertilization mediate maize yields across three soil types. Soil Till. Res..

[49] Zhou Z.H., Wang C.K., Luo Y.Q. (2020). Meta-analysis of the impacts of global change factors on soil microbial diversity and functionality. Nat. Commun..

[50] Zhang Z.H., Tariq A., Zeng F.J., Graciano C., Sun F., Chai X.T., Ahmed Z. (2021). Nitrogen and water addition regulate fungal community and microbial co-occurrence network complexity in the rhizosphere of Alhagi sparsifolia seedlings. Appl. Soil Ecol..

[51] Zhou J., Guan D.W., Zhou B.K., Zhao B.S., Ma M.C., Qin J., Jiang X., Chen S.F., Cao F.M., Shen D.L. (2015). Influence of 34-years of fertilization on bacterial communities in an intensively cultivated black soil in northeast China. Soil Biol. Biochem..

[52] Zhong Y., Yan W.M., Shangguan Z. (2015). Impact of long-term N additions upon coupling between soil microbial community structure and activity, and nutrient-use efficiencies. Soil Biol. Biochem..

[53] Luo G.W., Li L., Friman V.-P., Guo J.J., Guo S.W., Shen Q.R., Ling N. (2018). Organic amendments increase crop yields by improving microbe-mediated soil functioning of agroecosystems: A meta-analysis. Soil Biol. Biochem..

[54] Chau J.F., Bagtzoglou A.C., Willig M.R. (2011). The effect of soil texture on richness and diversity of bacterial communities. Environ. Forensics.

[55] Ma J.C., Ibekwe A.M., Yang C.H., Crowley D.E. (2016). Bacterial diversity and composition in major fresh produce growing soils affected by physiochemical properties and geographic locations. Sci. Total Environ..

[56] Xu A.X., Li L.L., Xie J.H., Zhang R.Z., Luo Z.Z., Cai L.Q., Liu C., Wang L.L., Anwar S., Jiang Y.J. (2022). Bacterial diversity and potential functions in response to long-term nitrogen fertilizer on the Semiarid Loess Plateau. Microorganisms.

[57] Farmer J., Zhang B., Jin X.X., Zhang P., Wang J.K. (2017). Long-term effect of plastic film mulching and fertilization on bacterial communities in a brown soil revealed by high through-put sequencing. Arch. Agron. Soil Sci..

[58] Zhang T.A., Chen H.Y., Ruan H.H. (2018). Global negative effects of nitrogen deposition on soil microbes. ISME J..

[59] Rousk J., Bååth E., Brookes P.C., Lauber C.L., Lozupone C., Caporaso J.G., Knight R., Fierer N. (2010). Soil bacterial and fungal communities across a pH gradient in an arable soil. ISME J..

[60] Wang H., Liu S.R., Zhang X., Mao Q.G., Li X.Z., You Y.M., Wang J.X., Zheng M.H., Zhang W., Lu X.K. (2018). Nitrogen addition reduces soil bacterial richness, while phosphorus addition alters community composition in an old-growth N-rich tropical forest in southern China. Soil Biol. Biochem..

[61] Bakker M.G., Schlatter D.C., Ottohanson L., Kinkel L.L. (2014). Diffuse symbioses: Roles of plant–plant, plant–microbe and microbe–microbe interactions in structuring the soil microbiome. Mol. Ecol..

[62] Hu Y., Chen M., Yang Z.L., Cong M.F., Zhu X.P., Jia H.T. (2022). Soil microbial community response to nitrogen application on a swamp meadow in the arid region of Central Asia. Front. Microbiol..

[63] Janssens I.A., Dieleman W., Luyssaert S., Subke J., Reichstein M., Ceulemans R., Ciais P., Dolman A.J., Grace J., Matteucci G. (2010). Reduction of forest soil respiration in response to nitrogen deposition. Nat. Geosci..

[64] Treseder K.K. (2008). Nitrogen additions and microbial biomass: A meta-analysis of ecosystem studies. Ecol. Lett..

[65] Zeng J., Liu X., Ling S., Lin X., Chu H. (2016). Nitrogen fertilization directly affects soil bacterial diversity and indirectly affects bacterial community composition. Soil Biol. Biochem..

[66] Xie Y., Wang Z.C., Cheng X.X., Qiu R.J., Hamoud Y.A., Hong C., Zong X.Y., Wang Y.s., Agathokleous E., Guo X.P. (2022). Dissecting the combined effects of cultivar, fertilization, and irrigation on rhizosphere bacterial communities and nitrogen productivity in rice. Sci. Total Environ..

[67] Dai Z.M., Su W.Q., Chen H.H., Barberán A., Zhao H.C., Yu M.J., Yu L., Brookes P.C., Schadt C.W., Chang S.X. (2018). Long-term nitrogen fertilization decreases bacterial diversity and favors the growth of Actinobacteria and Proteobacteria in agro-ecosystems across the globe. Global Chang Biol..

[68] Zhang Z.H., Tang G.L., Chai X.T., Liu B., Gao X.P., Zeng F.J., Wang Y., Zhang B. (2023). Different responses of soil bacterial and fungal communities in three typical vegetations following nitrogen deposition in an Arid Desert. Microorganisms.

[69] Ventura M., Canchaya C., Tauch A., Chandra G., Fitzgerald G.F., Chater K.F., Sinderen D.V. (2007). Genomics of Actinobacteria: Tracing the evolutionary history of an ancient phylum. Microbiol. Mol. Biol. R..

[70] Ai C., Liang G.Q., Sun J.W., Wang X.B., He P., Zhou W., He X.H. (2015). Reduced dependence of rhizosphere microbiome on plant-derived carbon in 32-year long-term inorganic and organic fertilized soils. Soil Biol. Biochem..

[71] Wei W., Yang M., Liu Y.X., Huang H.C., Ye C., Zheng J.F., Guo C.W., Hao M.W., He X.H., Zhu S.S. (2018). Fertilizer N application rate impacts plant-soil feedback in a sanqi production system. Sci. Total Environ..

[72] Guo Q.X., Yan L.J., Korpelainen H., Niinemets Ü., Li C.Y. (2019). Plant-plant interactions and N fertilization shape soil bacterial and fungal communities. Soil Biol. Biochem..

[73] Constancias F., Saby N.P.A., Terrat S., Dequiedt S., Horrigue W., Nowak V., Guillemin J.P., Biju-Duval L., Chemidlin Prévost-Bouré N., Ranjard L. (2015). Contrasting spatial patterns and ecological attributes of soil bacterial and archaeal taxa across a landscape. MicrobiologyOpen.

[74] Ma G., Kang J., Wang J.R., Chen Y.L., Lu H.F., Wang L.F., Wang C.Y., Xie Y.X., Ma D.Y., Kang G.Z. (2020). Bacterial community structure and predicted function in wheat soil from the North China Plain are closely linked with soil and plant characteristics after seven years of irrigation and nitrogen application. Front. Microbiol..

[75] Li H., Xu Z.W., Yang S., Li X.B., Top E.M., Wang R.Z., Zhang Y.G., Cai J.P., Yao F., Han X.G. (2016). Responses of soil bacterial communities to nitrogen deposition and precipitation increment are closely linked with aboveground community variation. Microb. Ecol..

[76] Wang Q., Wang C., Yu W.W., Ali T., Chen D.W., Huang Y., Ao J., Jiang Y., Huang Z. (2018). Effects of nitrogen and phosphorus inputs on soil bacterial abundance, diversity, and community composition in Chinese Fir Plantations. Front. Microbiol..

[77] Zhang H.F., Liu H.M., Zhao J.N., Li G., Lai X., Li J., Wang H., Yang D.L. (2018). Effects of simulated nitrogen deposition and precipitation change on soil bacterial community structure in a *Stipa baicalensis* steppe. Acta Ecol. Sinica.

[78] Li L., Song J., Peng C., Yang Z., Wang L., Lin J., Li L., Huang Z., Gong B. (2022). Co-occurrence network of microbes linking growth and immunity parameters with the gut microbiota in Nile tilapia (*Oreochromis niloticus*) after feeding with fermented soybean meal. Aquacult Rep.

[79] Qiu C., Bao Y., Petropoulos E., Wang Y., Zhong Z., Jiang Y., Ye X., Lin X., Feng Y. (2022). Organic and inorganic amendments shape bacterial indicator communities that can, in turn, promote rice yield. Microorganisms.

[80] Yang X.Y., Duan P.P., Wang K.L., Li D.J. (2023). Topography modulates effects of nitrogen deposition on soil nitrogen transformations by impacting soil properties in a subtropical forest. Geoderma.

[81] Miller M.N., Zebarth B.J., Dandie C.E., Burton D.L., Goyer C., Trevors J.T. (2008). Crop residue influence on denitrification, N_2_O emissions and denitrifier community abundance in soil. Soil Biol. Biochem..

[82] Li Y., Tremblay J., Bainard L.D., Cade-Menun B., Hamel C. (2020). Long-term effects of nitrogen and phosphorus fertilization on soil microbial community structure and function under continuous wheat production. Environ. Microbiol..

[83] Liao X.H., Tang T.G., Li J.N., Wang J.C., Neher D.A., Zhang W., Xiao J., Xiao D., Hu P.L., Wang K.L. (2024). Nitrogen fertilization increases the niche breadth of soil nitrogen-cycling microbes and stabilizes their co-occurrence network in a karst agroecosystem. Agric. Ecosyst. Environ..

[84] Liu C.R., Zhang Y.S., Liu H.R., Liu X.Q., Ren D.Y., Wang L., Guan D.H., Li Z.H., Zhang M.C. (2022). Fertilizer stabilizers reduce nitrous oxide emissions from agricultural soil by targeting microbial nitrogen transformations. Sci. Total Environ..

[85] Tang L., Zhong L., Xue K., Wang S.P., Xu Z.H., Lin Q.Y., Luo C.Y., Rui Y.C., Li X.Z., Li M. (2019). Warming counteracts grazing effects on the functional structure of the soil microbial community in a Tibetan grassland. Soil Biol. Biochem..

[86] Du R., Peng Y.Z., Ji J.T., Shi L.L., Gao R.T., Li X.C. (2019). Partial denitrification providing nitrite: Opportunities of extending application for anammox. Environ. Int..

[87] Hu X.J., Gu H.D., Liu J.J., Wei D., Zhu P., Cui X.A., Zhou B.K., Chen X.L., Jin J., Liu X.B. (2022). Metagenomics reveals divergent functional profiles of soil carbon and nitrogen cycling under long-term addition of chemical and organic fertilizers in the black soil region. Geoderma.

[88] Yang X.D., Tang S., Ni K., Shi Y.Z., Yi X.Y., Ma Q.X., Cai Y.J., Ma L.F., Ruan J.Y. (2023). Long-term nitrogen addition increases denitrification potential and functional gene abundance and changes denitrifying communities in acidic tea plantation soil. Environ. Res..

[89] Ren B.J., Wang W.Q., Shen L.D., Yang W.T., Yang Y.L., Jin J.H., Geng C.Y. (2023). Nitrogen fertilization rate affects communities of ammonia-oxidizing archaea and bacteria in paddy soils across different climatic zones of China. Sci. Total Environ..

[90] Zheng M.H., Zhou Z.H., Luo Y.Q., Zhao P., Mo J.M. (2019). Global pattern and controls of biological nitrogen fixation under nutrient enrichment: A meta-analysis. Glob. Change Biol..

[91] Shi X., Tan W., Tang S., Ling Q., Tang C., Qin P., Luo S., Zhao Y., Yu F., Li Y. (2023). Metagenomics reveals taxon-specific responses of soil nitrogen cycling under different fertilization regimes in heavy metal contaminated soil. J. Environ. Manage..

[92] Wang K., Flury M., Kuzyakov Y., Zhang H., Zhu W., Jiang R. (2025). Aluminum and microplastic release from reflective agricultural films disrupt microbial communities and functions in soil. J. Hazard. Mater..

[93] Zhao J., Ni T., Li J., Lu Q., Fang Z.Y., Huang Q.W., Zhang R.F., Li R., Shen B., Shen Q.R. (2016). Effects of organic–inorganic compound fertilizer with reduced chemical fertilizer application on crop yields, soil biological activity and bacterial community structure in a rice–wheat cropping system. Appl. Soil Ecol..

[94] Liu Z., Nan Z., Lin S., Meng W., Xie L., Yu H., Zhang Z., Wan S. (2024). Peanut-based intercropping systems altered soil bacterial communities, potential functions, and crop yield. Peer J..

[95] Li S., Yiminijiang A., Li R.X., Wu M.D., Long M.X., Yang P.Z., He S.B. (2025). Nutrient uptake and rhizosphere microbial community as related to yield advantage in broomcorn millet—alfalfa intercropping under different row configurations. BMC Plant Biol..

