# Supplementary material for: Nitrogen Fertilization Effects on Soil Bacterial Communities, Nitrogen-Cycling Genes, and Wheat Yield Across Different Soil Types in the North China Plain
Source: Microorganisms. 2025 Oct 15;13(10):2382. doi: 10.3390/microorganisms13102382 (PMC12566270; doi:10.3390/microorganisms13102382)
Supplement: Supplementary file 1 [file microorganisms-13-02382-s001.zip › microorganisms-3860279-supplementary.pdf]

Nitrogen Fertilization Effects on Soil Bacterial Communities, Nitrogen-Cycling Genes, and Wheat Yield Across Different Soil Types in the North China Plain

Geng Ma [1], Xiaoyan Zhang [2], Xiaojie Han [2], Juan Kang [1], Haiyan Zhang [1], Yanfei Zhang [1],

1 College of Agronomy, Henan Agricultural University, Zhengzhou 450002, China
2 College of Resource and Environmental Sciences, Henan Agricultural University, Zhengzhou 450002, China
3 National Engineering Research Centre for Wheat, Henan Agricultural University, Zhengzhou 450002, China
* Correspondence: xmzxwang@163.com (C. W.)

Table S1. Basic soil properties from three soil styles of experimental sites at the start of the field experiment

Soil	pH	SOM (g kg^{-1})	TN (g kg^{-1})	AN (mg kg^{-1})	AP (mg kg^{-1})	AK (mg kg^{-1})	Clay (%)	Silt (%)	Sand (%)
FS	7.68	17.23	1.02	98.58	20.53	380.6	21.91	32.26	45.83
SS	7.51	8.32	0.53	73.66	7.43	154.3	14.52	27.61	57.87
BS	7.12	24.81	1.32	128.39	18.6	301.2	40.25	47.33	12.42

Table S2. Effects of N fertilization regimes on soil properties in three soil styles

Soil	N rate	Moisture (%)	pH	OM (g kg^{-1})	TN (g kg^{-1})	AN (mg kg^{-1})	AP (mg kg^{-1})	AK (mg kg^{-1})	NO_3^--N (g kg^{-1})
FS	N0	19.11g	7.72a	17.43e	0.88d	97.98e	19.93cd	362.58b	15.98f
	N1	21.10e	7.74a	20.77d	1.06c	114.31d	21.39bc	392.42a	44.82a
	N2	20.13f	7.44b	19.34f	1.08c	112.20d	23.98a	359.93b	41.68a
	N3	20.36ef	7.25def	19.28f	1.05c	111.68d	16.85e	407.72a	31.88c
SS	N0	12.24h	7.65a	8.02h	0.48e	76.38g	14.51f	152.04f	11.27g
	N1	9.98i	7.32cd	6.59i	0.42e	73.74g	9.98h	95.81g	21.17e
	N2	9.42i	7.22def	7.52hi	0.49e	74.27g	12.16g	151.41f	26.78d
	N3	9.78i	7.09g	6.92hi	0.47e	83.23f	15.06ef	191.43e	36.56b
BS	N0	25.30c	7.28cde	27.16a	1.42a	132.74b	19.75cd	360.95a	18.70c
	N1	26.77b	7.17f	22.79d	1.29b	126.43c	18.79d	333.33c	25.63d
	N2	23.40d	7.02g	24.01c	1.36b	135.38b	19.15d	347.59c	28.60cd
	N3	28.31a	6.88h	25.27b	1.32b	143.81a	22.13b	287.34d	41.71a

[1] OM: organic matter; TN: total nitrogen; AN: Available nitrogen; AP: available phosphorus; AK: available. Values are mean in four replicates with different letters in a column for the three soil types indicate significant difference among all treatments ($p < 0.05$).

Table S3. Spearman's rank correlation coefficients between diversity indices and soil properties for three soils (n=16)

Soil	Diversity index	Moisture (%)	pH (g kg^{-1})	OM (g kg^{-1})	TN (mg kg^{-1})	AN (mg kg^{-1})	AP (mg kg^{-1})	AK (mg kg^{-1})	NO_3^--N (g kg^{-1})
FS	OTUs	-0.068	-0.118	-0.012	-0.226	-0.178	-0.512*	0.106	0.038
	Shannon	-0.056	0.100	0.006	-0.194	-0.395	-0.432	-0.044	-0.012
	Chao	0.062	0.038	0.038	0.068	-0.100	-0.356	0.053	-0.035
	Ace	0.091	0.211	0.150	0.015	-0.276	-0.444	0.071	0.115
SS	OTUs	0.553*	0.705**	0.321	0.037	-0.245	0.121	-0.291	-0.849***
	Shannon	0.118	0.237	0.176	0.243	-0.330	0.174	-0.229	-0.428
	Chao	0.418	0.727**	0.338	0.043	-0.347	-0.024	-0.456	-0.852***
	Ace	0.459	0.772***	0.382	-0.003	-0.35	-0.044	-0.494	-0.884***
BS	OTUs	-0.003	-0.224	-0.382	-0.035	-0.135	-0.294	-0.250	-0.047
	Shannon	-0.112	-0.052	-0.203	0.121	-0.184	-0.350	-0.085	-0.185
	Chao	-0.168	0.004	-0.026	0.368	-0.130	-0.321	0.103	-0.206
	Ace	-0.038	-0.081	-0.324	0.066	-0.200	-0.226	-0.159	-0.074

Significant levels: $^{*}P$<0.05.$^{**}P$<0.01 and $^{***}P$<0.001.

Table S4. Spearman's correlation coefficients between diversity indices and soil properties for three soils

Genes	FS					SS					BS				
	RB41	*Bacillus*	*Sphingomonas*	*Gaiella*	*Streptomyces*	RB41	*Bacillus*	*Sphingomonas*	*Gaiella*	*Streptomyces*	RB41	*Bacillus*	*Sphingomonas*	*Gaiella*	*Streptomyces*
nirB	-0.831**	0.616*	0.548*	0.461	0.573*	-0.613*	0.031	0.473	0.353	0.384	-0.560*	0.525*	0.689**	0.437	0.792**
nirD	0.701**	-0.622*	-0.426	-0.589*	-0.268	0.694**	-0.413	-0.306	-0.501*	-0.399	0.683**	-0.381	0.227	-0.707**	-0.078
narL	-0.844**	0.304	0.231	0.714**	0.803**	-0.568*	-0.195	0.698**	-0.014	0.480	0.155	0.504*	0.825**	0.107	0.571*
nrfA	-0.140	0.735**	0.333	-0.191	-0.367	-0.537*	0.803**	0.008	0.372	0.009	-0.340	0.421	-0.200	0.136	0.365
nrfH	-0.231	0.836**	0.282	-0.130	-0.255	-0.519*	0.798**	-0.047	0.433	0.037	-0.443	0.629**	-0.015	0.198	0.510*
nasA	-0.757**	0.524*	0.458	0.456	0.648**	-0.077	-0.211	0.132	0.093	0.088	-0.163	0.463	0.872**	0.164	0.767**
narB	-0.304	0.501*	0.668**	-0.230	0.062	-0.753**	0.417	0.538*	0.205	0.275	0.130	0.044	0.746**	0.052	0.459
nasB	-0.454	-0.186	-0.269	0.479	0.670**	0.069	-0.247	0.226	-0.146	0.136	-0.561*	0.507*	0.664**	0.230	0.895**
nirA	-0.641**	0.824**	0.457	0.316	0.127	-0.772**	0.635**	0.277	0.603*	0.335	0.003	0.363	0.474	0.516*	0.105
nirK	-0.622*	0.490	0.687**	0.191	0.523*	-0.577*	-0.059	0.668**	-0.021	0.405	-0.194	0.533*	0.737**	0.043	0.823**
norB	-0.205	0.212	0.821**	-0.033	0.145	-0.447	-0.273	0.682**	0.124	0.461	0.088	0.224	0.575*	-0.336	0.710**
norC	-0.525*	0.730**	0.708**	0.193	0.088	-0.862**	0.150	0.624**	0.760**	0.671**	-0.138	-0.181	-0.115	-0.254	0.117
narG	-0.856**	0.367	0.247	0.708**	0.743**	-0.627**	-0.226	0.731**	0.095	0.540*	0.119	0.532*	0.821**	0.152	0.563*
narH	-0.831**	0.389	0.235	0.563*	0.649**	-0.656**	-0.166	0.743**	0.117	0.526*	0.037	0.512*	0.693**	0.186	0.498*
napA	-0.651**	0.758**	0.734**	0.258	0.243	-0.838**	0.233	0.655**	0.448	0.515*	-0.106	0.112	0.572*	-0.006	0.546*
napB	-0.651**	0.748**	0.742**	0.255	0.243	-0.829**	0.187	0.710**	0.363	0.476	-0.121	0.131	0.566*	-0.004	0.553*
nosZ	-0.289	0.572*	0.737**	-0.222	-0.060	-0.778**	0.271	0.652**	0.253	0.367	-0.144	0.545*	0.781**	0.206	0.727**
nirS	0.536*	-0.224	0.095	-0.496	-0.230	-0.623**	0.216	0.527*	0.300	0.493	-0.053	-0.266	-0.391	-0.233	-0.229
nifD	-0.377	0.516*	0.576*	-0.054	-0.113	-0.776**	0.093	0.761**	0.204	0.399	-0.194	0.042	-0.063	-0.278	0.309
nifH	-0.303	0.528*	0.555*	-0.126	-0.235	-0.784**	0.107	0.755**	0.213	0.402	-0.198	0.000	-0.087	-0.268	0.307
anfG	-0.397	0.021	0.497	0.210	0.377	-0.448	-0.404	0.768**	-0.081	0.369	-0.228	0.242	0.730**	-0.105	0.766**
nifK	-.378	0.515*	0.578*	-0.053	-0.111	-0.776**	0.093	0.761**	0.204	0.399	-0.194	0.042	-0.063	-0.278	0.309
hao	0.068	-0.440	-0.080	0.174	0.425	-0.281	-0.534*	0.546*	0.121	0.551*	-0.312	0.382	0.232	0.288	0.323
pmoA-amoA	0.001	-0.333	0.002	0.296	0.413	-0.376	-0.459	0.587*	0.186	0.589*	-0.227	0.407	0.335	0.251	0.398
PmoB-amoB	0.001	-0.333	0.002	0.296	0.413	-0.376	-0.459	0.587*	0.186	0.589*	-0.227	0.407	0.335	0.251	0.398
PmoC-amoC	-0.035	-0.268	0.008	0.343	0.394	-0.408	-0.472	0.632**	0.176	0.598*	-0.301	0.410	0.292	0.276	0.399

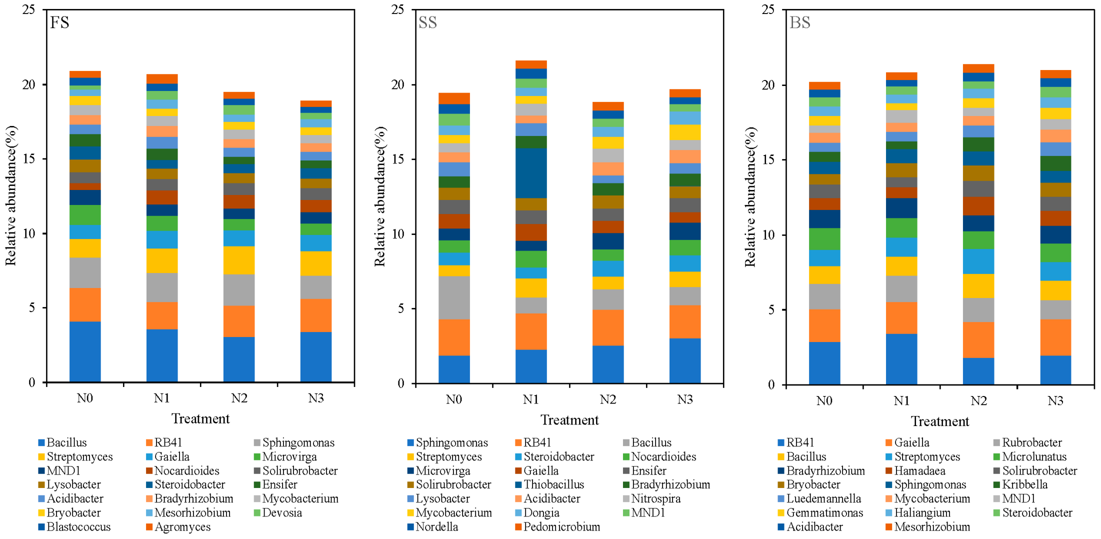

Figure S1. Relative abundance of top 20 bacterial groups (genus level) under different N treatments in three soil types.

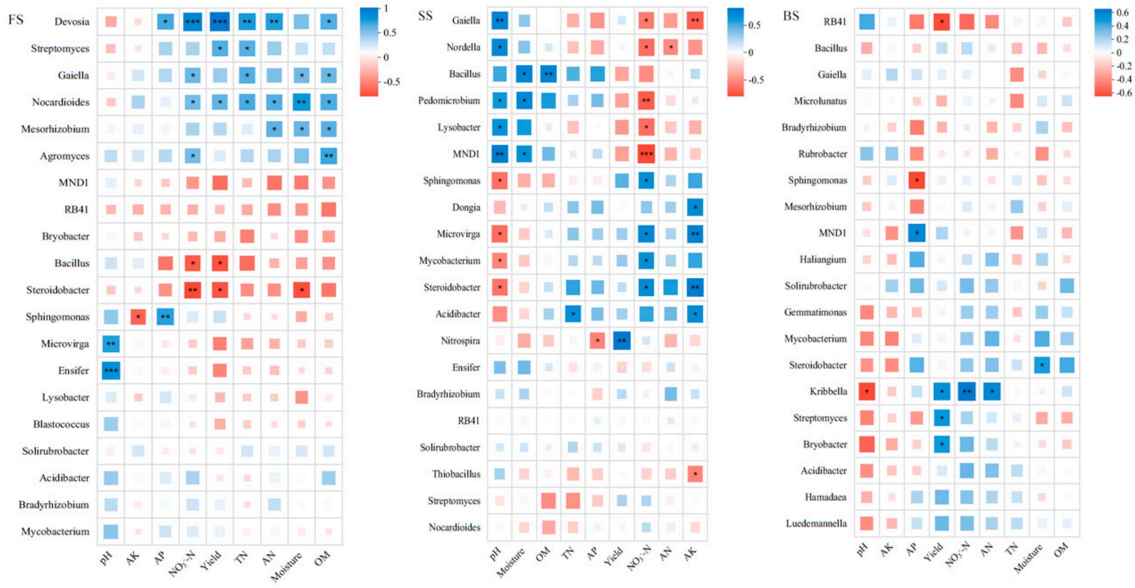

Figure S2. Pearson correlation heatmap between bacterial genera and soil properties in FS, SS, and BS. R-values are displayed in different colors, as indicated by the color code on the right of the heatmap. Significance levels are denoted by *$p < 0.05$, **$p < 0.01$, and ***$p < 0.001$.

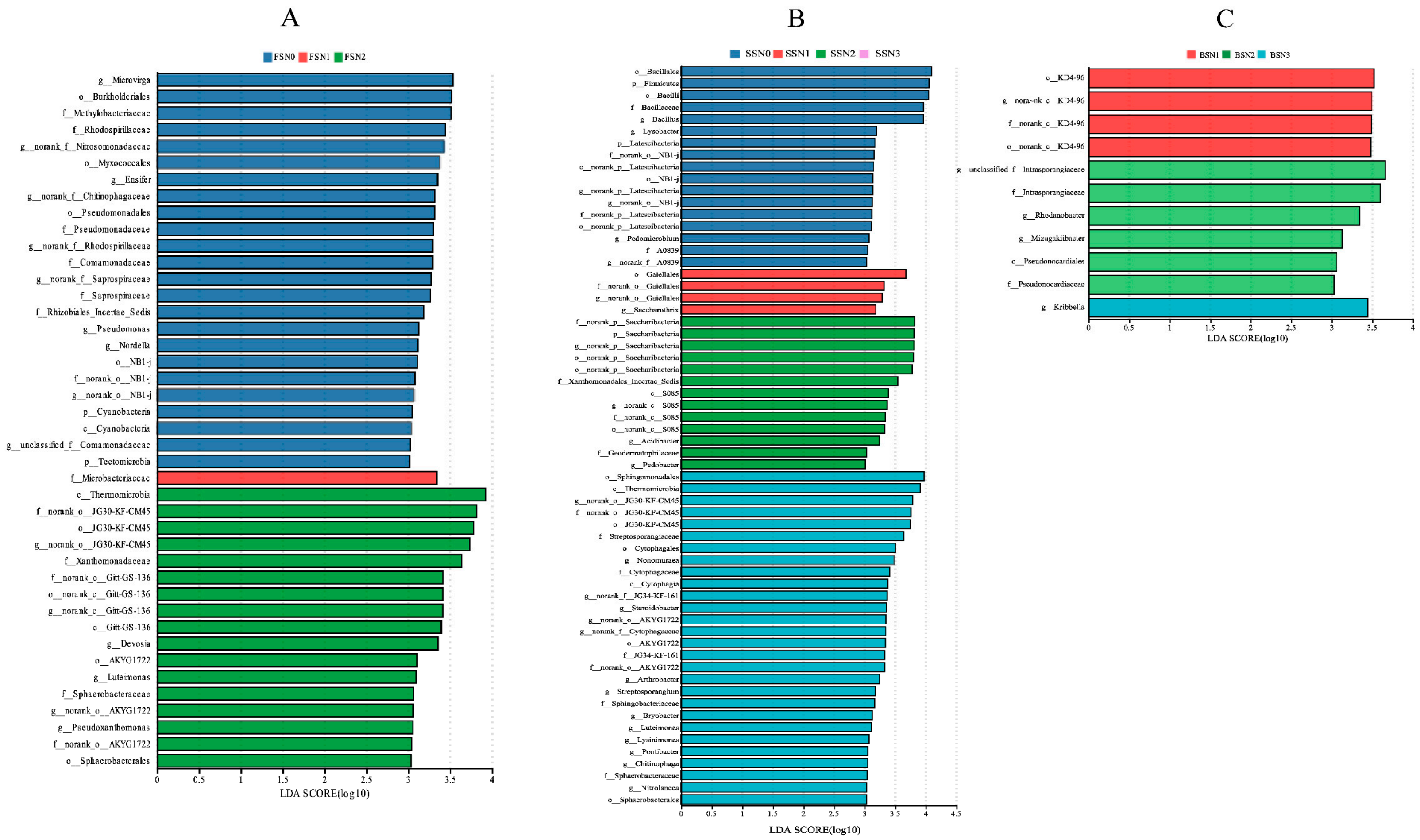

Figure S3. Indicator bacteria with LDA scores of 3 or greater in bacterial communities from the four N treatments in FS (A), SS (B), BS (C).

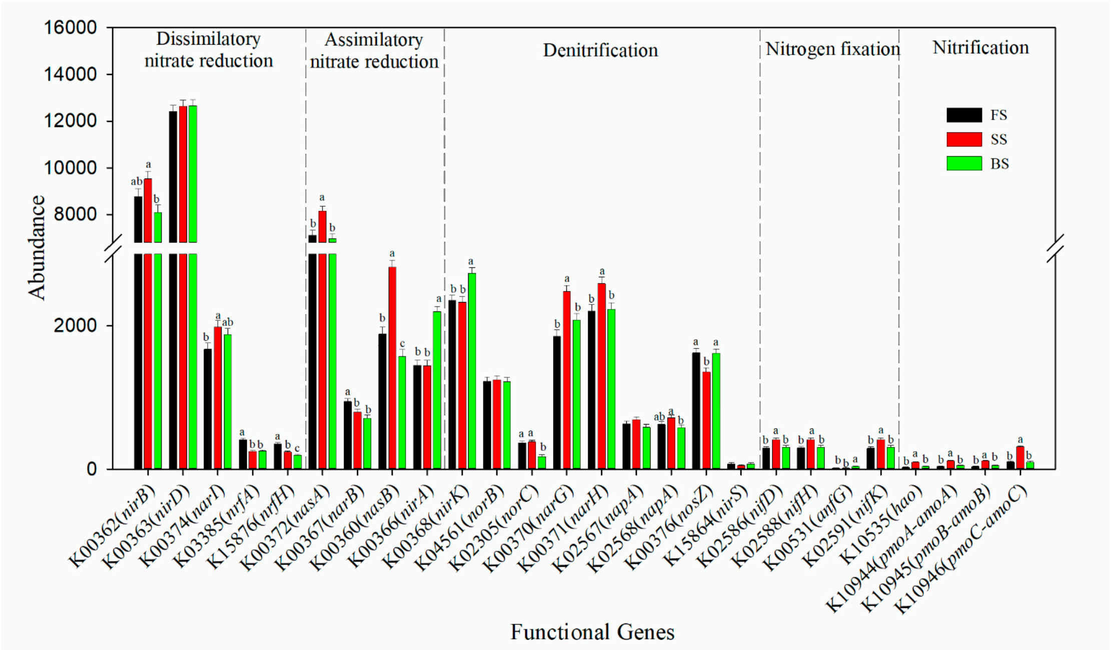

Figure S4. Abundance of selected orthologs associated with nitrogen cycle in FS, SS, and BS, derived from PICRUSt2 analysis. Bars with different letters are significantly different ($p < 0.05$).
